# Comprehensive molecular and phenotypic profiling of uropathogenic *Escherichia coli* in a Honduran healthcare setting: virulence, resistance and phylogeny

**DOI:** 10.3389/fmicb.2025.1656938

**Published:** 2025-10-02

**Authors:** Bryan Ortiz, Manuel G. Ballesteros-Monrreal, Celeste Galindo, Daniel Rivera, Luis Rivera, Fernando Pérez, Victoria Maldonado, Elixia Valle, Ana Estrada, Pablo Mendez-Pfeiffer, Lourdes Enríquez, Dora Valencia, Gustavo Fontecha

**Affiliations:** ^1^Instituto de Investigaciones en Microbiología, Facultad de Ciencias, Universidad Nacional Autónoma de Honduras, Tegucigalpa, Honduras; ^2^Departamento de Ciencias Químico-Biológicas y Agropecuarias, Universidad de Sonora, Hermosillo, Mexico; ^3^Instituto Hondureño de Seguridad Social, Tegucigalpa, Honduras; ^4^Departamento de Bionanotecnología, Centro de Nanociencias y Nanotecnología, Universidad Nacional Autónoma de México, Km 107 Carretera Tijuana-Ensenada, Ensenada, Mexico

**Keywords:** Uropathogenic *Escherichia coli* (UPEC), UPEC virulence, UPEC resistance, Honduras, urinary infection

## Abstract

**Introduction:**

Urinary tract infections (UTIs) are a major public health concern, further complicated by the rise of multidrug-resistant bacterial strains. Uropathogenic *Escherichia coli* (UPEC) is the primary causative agent of UTIs, notable for its genetic diversity and its ability to acquire both virulence and antimicrobial resistance determinants.

**Methods:**

This study conducted a comprehensive phenotypic and molecular characterization of 126 UPEC strains isolated from a tertiary care hospital in Tegucigalpa, Honduras. Seventeen virulence genes were screened, antimicrobial susceptibility to 17 antibiotics was assessed, phylogenetic grouping was performed, and potential clonal relationships were analyzed using ERIC-PCR.

**Results:**

Strains isolated from male patients exhibited significantly higher virulence gene counts (mean: 10.48 vs. 8.06; *p* = 0.0029), resistance indices (RI = 0.46 vs. 0.27; *p* < 0.0001), and multidrug resistance rates (88% vs. 63%; *p* = 0.009) compared to those from female patients. Extended-spectrum *β*-lactamase (ESBL) production was observed in 42% of isolates, with a higher prevalence in males (59%; *p* = 0.049). Phylogroup B2 was the most frequent (29%) and was significantly associated with virulence genes *papG-II*, *hlyA*, *cnf-1*, *fyuA*, and *iucD*. Despite high genetic heterogeneity observed through ERIC-PCR, clonal clusters sharing similar phylogroups, virulence profiles, and resistance phenotypes were identified. A weak but significant correlation was found between virulence and resistance indices (r = 0.1796; *p* = 0.0442).

**Discussion:**

This study provides the first in-depth molecular and phenotypic characterization of UPEC in Honduras. The detection of highly virulent and multidrug-resistant strains underscores the need to reinforce local molecular surveillance and to revise empirical treatment guidelines based on local epidemiological data.

## 1 Introduction

Urinary tract infections (UTIs) are a major global public health concern, affecting over 150 million individuals each year (Öztürk and Murt, 2020). These infections are associated with considerable morbidity and encompass a broad spectrum of clinical manifestations, ranging from asymptomatic bacteriuria and cystitis to severe pyelonephritis, which may progress to septic shock and potentially fatal multiorgan failure (Agarwal et al., 2012; McLellan and Hunstad, 2016). In 2019 alone, an estimated 400 million UTI cases were reported worldwide, resulting in over 230,000 deaths and 520,200 disability-adjusted life years (DALYs)—a nearly 70% increase compared to 1990 (Yang et al., 2022).

*Escherichia coli* is a Gram-negative bacterium that typically resides as a commensal in the gastrointestinal tracts of humans and animals (Lagerstrom and Hadly, 2023; Lagerstrom and Hadly, 1948). However, its high genomic plasticity enables it to acquire exogenous genetic elements, such as plasmids, transposons, insertion sequences, and integrons (Yousuf et al., 2020; Tenaillon et al., 2010). This capacity promotes the emergence of strains with enhanced virulence and antimicrobial resistance, posing a significant threat to public health (Lagerstrom and Hadly, 1948; Whelan et al., 2023). Based on infection site, clinical presentation, and genotypic traits, *E. coli* strains are broadly classified into two categories: intestinal pathotypes, which cause diarrheal disease, and extraintestinal pathotypes (Regua-Mangia et al., 2010). The latter include uropathogenic *E. coli* (UPEC), neonatal meningitis-associated *E. coli* (NMEC), sepsis-associated *E. coli* (SEPEC), avian pathogenic *E. coli* (APEC), and mammary pathogenic *E. coli* (MPEC) (Sora et al., 2021).

*E. coli* strains are currently categorized into eight major phylogenetic groups (A, B1, B2, C, D, E, F, and G), which reflect their evolutionary relationships and pathogenic potential. In addition, five cryptic clades (I–V) have been described, corresponding to phylogenetically related but distinct *Escherichia* species (Clermont et al., 2000; Clermont et al., 2013; Clermont et al., 2019). Most extraintestinal pathogenic strains belong to phylogroups B2 and D (Halaji et al., 2022), whereas commensal strains are more commonly associated with phylogroups A and B1 (da Silva et al., 2017).

UPEC is the predominant cause of UTIs in both community and hospital settings, accounting for approximately 80% of community-acquired and over 50% of healthcare-associated infections (Kang et al., 2018; Xia et al., 2017; Kucheria et al., 2005). It is responsible for more than 75% of uncomplicated UTIs and about 65% of complicated cases (Whelan et al., 2023; Medina and Castillo-Pino, 2019). This high prevalence is closely linked to the virulence potential of phylogroups B2 and D, which harbor strains capable of expressing a wide range of virulence factors (Halaji et al., 2022; Royer et al., 2023). These include adhesins, toxins, iron acquisition systems, and immune evasion mechanisms, all of which facilitate adherence, invasion, and persistence within the urinary tract epithelium, contributing to recurrent and difficult-to-treat infections (Alam Parvez and Rahman, 2018; Behzadi and Carevic, 2019; Ballesteros-Monrreal et al., 2021).

In addition to their pathogenicity, UPEC strains often exhibit multidrug resistance (MDR), phenotypes, representing a growing public health challenge (Akhand and Karicheri, 2022). MDR infections are projected to cause an increasing number of deaths globally by 2050 (de Kraker et al., 2016). Furthermore, as with virulence traits, antimicrobial resistance profiles in UPEC vary considerably by geographic region, complicating empirical treatment decisions (Yang et al., 2022; Ballesteros-Monrreal et al., 2021; Ramakrishnan et al., 2022). This highlights the urgent need for local and regional epidemiological studies to better understand UPEC’s resistance and virulence patterns, thereby informing and updating clinical management guidelines.

To date, no epidemiological surveillance study in Honduras has addressed the molecular characterization of UPEC in clinical settings. Therefore, this study aimed to characterize the antimicrobial resistance, virulence gene distribution, and phylogenetic profiles of UPEC isolates recovered from a major tertiary hospital in Tegucigalpa, Honduras. These findings are intended to support clinicians and public health stakeholders by providing data for more effective management of UTIs in the region.

## 2 Materials and methods

### 2.1 Clinical isolates

Urine cultures phenotypically identified as *Escherichia coli* with colony counts ≥100,000 CFU/mL, processed between July and August 2022 at a tertiary-level hospital of the Honduran Social Security Institute (IHSS) in Tegucigalpa, were donated to this study. The isolates were provided along with anonymized sociodemographic data (sex, age, and hospitalization status). No additional clinical information, such as treatment history, underlying diseases, or urinary cathether use, was available. Only molecular characterization of the isolates was performed. No direct contact with patients occurred at any stage of the study.

### 2.2 Phenotypic identification and antimicrobial susceptibility testing

Bacterial identification was performed using the BD Phoenix™ Identification System for Gram-negative bacilli, following the manufacturer’s instructions. Briefly, one to two colonies from a 24-h culture were suspended in Phoenix ID broth to a 0.5 McFarland turbidity standard. From this standardized suspension, 25 μL were transferred to Phoenix AST broth, which was supplemented with a drop of Phoenix AST indicator (containing methylene blue and resazurin) to detect bacterial growth. The mixture was then loaded into BD Phoenix™ NMIC-500 panels (catalog number BD 449023), which were sealed, registered, and processed using the BD Phoenix™ M50 system. Results were analyzed using Epicenter data management software (version 6.61A, BD Diagnostic Systems) after incubation for 6 to 16 h.

Antibiotics were grouped into 12 categories, according to the classification proposed by Magiorakos et al. (2012) (1) Aminoglycosides: Amikacin (AMK), Gentamicin (GM) (2) Sulfonamides: Trimethoprim/Sulfamethoxazole (TSX); (3) Nitrofurans: Nitrofurantoin (MAC); (4) Fluoroquinolones: Ciprofloxacin (CIP), Levofloxacin (LVX); (5) Penicillins with *β*-lactamase inhibitors: Amoxicillin/Clavulanic acid (AMC), Piperacillin/Tazobactam (TZP); (6) Penicillins: Ampicillin (AMP); (7) First-generation cephalosporins: Cefuroxime (CFX); (8) Second-generation cephalosporins: Cefazolin (CZ); (9) Third-generation cephalosporins: Ceftriaxone (CRO); (10) Fourth-generation cephalosporins: Cefepime (FEP); (11) Cephamycin: Cefoxitin (FOX); and (12) Carbapenems: Ertapenem (ETP), Imipenem (IMP), Meropenem (MEM). Based on the resistance profile of each isolate, strains were classified as follows: Non-multidrug-resistant (NMDR): Non-susceptible to two or fewer categories; Multidrug-resistant (MDR): Non-susceptible to three or more categories; extensively drug-resistant (XDR): Susceptible to only one of the tested categories; Pandrug-resistant (PDR): Non-susceptible to all tested categories (Magiorakos et al., 2012).

The prevalence of resistance for each antibiotic was graphically represented as a percentage. Additionally, a resistance index (*RI*) was calculated for each isolate using the formula previously reported by Karim et al. (2023),


$$\mathrm{RI}=\frac{a}{\;b}$$

Where “*a*” represents the number of antibiotics to which a given isolate was resistant, and “*b*” corresponds to the total number of antibiotics tested in this study. The resulting *RI* values were subsequently used for comparative analyses based on patient sex, age group, phylogroup classification, and other variables.

### 2.3 DNA extraction and molecular identification

Phenotypically confirmed *E. coli* isolates were inoculated in Luria-Bertani (LB) broth (Liofilchem®) and incubated at 37 °C for 24 h. Genomic DNA was extracted using the Wizard® Genomic DNA Purification Kit (Promega, Madison, WI, USA), following the manufacturer’s protocol. DNA extracts were eluted in 100 μL of elution buffer and stored at −20 °C until further use.

Molecular identification of *E. coli* was performed by PCR amplification of the *ybbW* gene, which encodes an allantoin receptor reported as highly specific to this species (Walker et al., 2017). PCR reactions were conducted in a final volume of 25 μL containing 12.5 μL of 2 × PCR Master Mix (Promega Corp. Madison, WI, USA), 1 μL of each primer (10 μM), 8 μL of nuclease-free water, and 1.5 μL of DNA (40 ng/μL). Thermocycling conditions were as follows: initial denaturation at 95 °C for 4 min; 37 cycles of denaturation at 95 °C for 70 s, annealing at 60 °C for 70 s, and extension at 72 °C for 70 s; followed by a final extension step at 72 °C for 10 min. All PCR products were resolved on 2% agarose gels stained with ethidium bromide and visualized using the Gel Doc™ EZ Imaging System. Primer sequences used for amplification are listed in Supplementary Table 1.

### 2.4 Virulence gene profiling

Seventeen virulence genes associated with UPEC pathogenesis in the urinary tract were analyzed. These genes were functionally grouped into five categories: (1) Adhesion: *fimH*, *papG-II*, *papC*, *sfaS/focC*; (2) Motility: *fliCD*; (3) Toxin production: *hlyA*, *cnf*-1, *vat*, *sat*; (4) Immune evasion: *agn43*, *traT*, *kpsM;* and (5) Iron acquisition: *feoB*, *fyuA*, *iucD*, *iha*, *iutA.* Gene detection was performed via multiplex PCR following the protocol described by Ballesteros-Monrreal et al. (2021). PCR reactions were carried out in a final volume of 25 μL, containing 12.5 μL of 2 × PCR Master Mix (Promega Corp. Madison, WI, USA), 1 μL of each primer (10 μM), and 2 μL of genomic DNA. Positive controls included DNA from *E. coli* strains CFT073, ATCC 25922, and UPEC 39, as described in prior publications (Ballesteros-Monrreal et al., 2021). Primer sequences, amplicon sizes, and annealing temperatures (Tm, °C) are provided in Supplementary Table 1.

### 2.5 Phylogenetic group determination

Phylogenetic group assignment was performed using Clermont et al. (2000, 2013, 2019) methods, based on PCR detection of the *arpA*, *chuA*, *yjaA*, *trpA*, *ybgD*, and *cfaB* genes, as well as the TspE4. C2 DNA fragment. PCR reactions were conducted following previously described protocols (Mendoza et al., 2023).

### 2.6 Eric PCR and clonality estimation

Clonality among all isolates was assessed through the analysis of consensus sequences of repetitive intergenic regions (ERIC), a technique widely validated and recommended for molecular typing of *E. coli* (Alsultan and Elhadi, 2022; López-Ramírez et al., 2018; Dishan et al., 2023; Ardakani and Ranjbar, 2016; Mahmoud et al., 2020). The methodology followed that described by Movahedi et al. (2021). PCR reactions were carried out in a final volume of 25 μL containing 12.5 μL of 2 × PCR Master Mix (Promega Corp., Madison, WI, USA), 8.5 μL of nuclease-free water, 2 μL of genomic DNA, and 1 μL of each ERIC primer. Thermocycling conditions were as follows: initial denaturation at 94 °C for 5 min; 40 cycles of denaturation at 94 °C for 1 min, annealing at 53 °C for 40 s, and extension at 72 °C for 1 min; followed by a final extension at 72 °C for 5 min. Band sizes, in relation to the molecular weight marker, as well as the similarity matrix for dendrogram construction, were generated using the GelJ v2.0 software.^1^ Cluster analysis was performed using the unweighted pair group method with arithmetic mean (UPGMA) and the DICE similarity coefficient. To avoid overestimation of clonality, isolates were classified as “suspected clones” only when they shared ≥90% similarity in ERIC-PCR patterns and exhibited identical or highly similar resistance, virulence, and phylogroup profiles. This integrative approach ensured a more stringent and reliable interpretation of genetic relatedness. Clonal distribution was further analyzed in relation to hospital wards and patient origin (hospitalized vs. non-hospitalized).

### 2.7 Statistical analyses and graphical representations

A comprehensive statistical analysis approach was employed to ensure the reliability and interpretability of results. Descriptive statistics were initially calculated, including means, medians, and interquartile ranges, according to the distribution of the data. Assumptions of normality and homogeneity of variances were evaluated using the Shapiro–Wilk and Bartlett tests, respectively. Due to violations of these assumptions in some groups, the non-parametric Kruskal–Wallis test was used for overall group comparisons. When significant differences were observed, *post hoc* analyses were conducted using Dunn’s test with Bonferroni correction to determine which specific groups differed. For categorical variables such as sex, phylogenetic group, or gene presence/absence, Fisher’s exact test was used. Additionally, odds ratios (ORs) and relative risks (RRs) with 95% confidence intervals were calculated to assess the strength of associations between categorical variables. To explore potential associations between virulence factors and antimicrobial resistance, Pearson correlation analysis was performed. A *p* < 0.05 was considered statistically significant in all analyses.

All statistical analyses were performed using GraphPad Prism version 7.0 (GraphPad Software, San Diego, CA, USA)^2^ and JASP v0.19.3.^3^ Graphical representations were generated using GraphPad Prism and RStudio with R version 4.5.0 (2025-04-11). Data visualization in R was conducted using the ggplot2, pheatmap, and magick packages (R Core Team, 2025). ERIC-PCR dendrograms were visualized and edited using the interactive iTOL environment, version 7.2 (Letunic and Bork, 2021).

## 3 Results

### 3.1 Patients and clinical isolates

Between July and August 2022, a total of 126 clinical isolates were identified as *Escherichia coli* through both biochemical and molecular methods. Of these, 99 isolates (78.6%) were obtained from female patients, whose ages ranged from 0 to 88 years, while the remaining 27 isolates (21.4%) were from male patients aged between 0 and 85 years.

Analysis of age group distribution revealed that the 13–50 years category was the most prevalent among both sexes, accounting for 41% of female and 59% of male patients. This was followed by patients older than 50 years, comprising 38% of females and 22% of males. However, no statistically significant association was found between age groups and patient sex (*p* > 0.05).

### 3.2 Virulence genes

#### 3.2.1 Virulence genes by sex and age group

Following molecular confirmation of *E. coli* isolates, the presence of 17 virulence-associated genes was assessed. Isolates from female patients carried an average of 8.06 ± 3.26 virulence genes (range: 3–15), while those from male patients showed a significantly higher average of 10.48 ± 2.69 genes (range: 7–15). This difference was statistically significant (*p* = 0.0029), suggesting a higher virulence gene burden in isolates from male patients.

When stratified by age group, isolates from females under the age of five harbored the highest average number of virulence genes (9.43 ± 3.85), although this trend did not reach statistical significance (*p* > 0.05). Among male patients, the 6–12 years age group showed the highest virulence gene load, with an average of 13.3 ± 3.05 genes, a statistically significant difference when compared to the 13–50 and >50 years age groups (*p* < 0.05).

**Figure 1:**
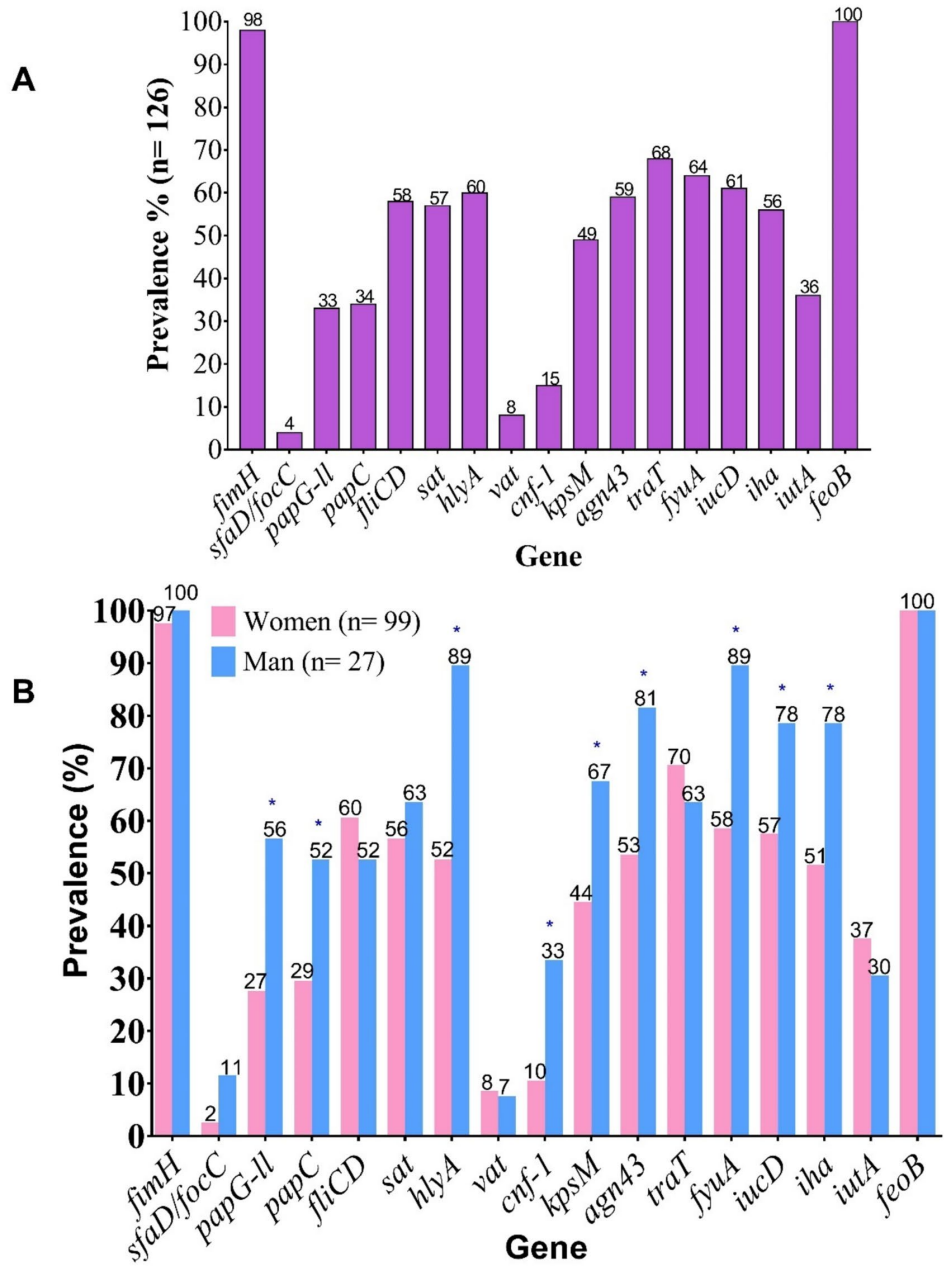
Prevalence of virulence-associated genes (VAGs) in clinical UPEC isolates. **(A)** Overall prevalence of VAGs. **(B)** Prevalence of VAGs stratified by patient sex. *fimH*, Fimbrial adhesin of type 1 pilus; *sfaD/focC*, S fimbriae minor subunit / F1C fimbriae chaperone; *papG*-II, Type P pilus adhesin allele 2; *papC*, Type P pilus chaperone; *fliCD*, Flagellin subunit / flagellar cap; *sat*, Autotransporter secreted toxin; *hlyA*, α-hemolysin; *vat*, Vacuolating autotransporter toxin; *cnf*-1, Cytotoxic necrotizing factor 1; *kpsM*, Capsular antigen variant; *agn43*, Antigen 43; *traT*, Serum resistance protein; *fyuA*, Yersiniabactin receptor; *iucD*, Aerobactin biosynthesis gene; *iha*, Bifunctional enterobactin receptor/adhesin protein; *iutA*, Ferric aerobactin receptor; *feoB*, Ferrous iron transport protein B.

Regarding the prevalence of individual genes, the most frequently detected across both sexes were the type 1 fimbrial adhesin gene *fimH* (96%) and the iron acquisition gene *feoB* (100%) (Figure 1A). Notably, a higher overall prevalence of virulence genes was observed in isolates from male patients. However, statistically significant differences (*p* < 0.05) were observed only for the following genes: *papG-II*, *papC*, *hlyA*, *cnf-1*, *kpsM*, *fyuA*, *iucD*, and *iha* (Figure 1B), which may indicate a potential sex-specific association of certain virulence factors with infections in male patients.

#### 3.2.2 Virulence genes and clinical setting

Potential differences in the distribution of virulence genes according to the clinical origin of the isolates—hospital-acquired vs. community-acquired—were investigated. Although no statistically significant differences were found in the overall prevalence of virulence genes between these patient groups (*p* > 0.05) (Supplementary Figure 1), noteworthy pathogenic trends emerged. Isolates from outpatients more frequently carried genes associated with iron acquisition, serum resistance, and renal adherence, whereas hospital-acquired isolates exhibited a higher frequency of genes linked to motility and toxigenicity.

**Figure 2:**
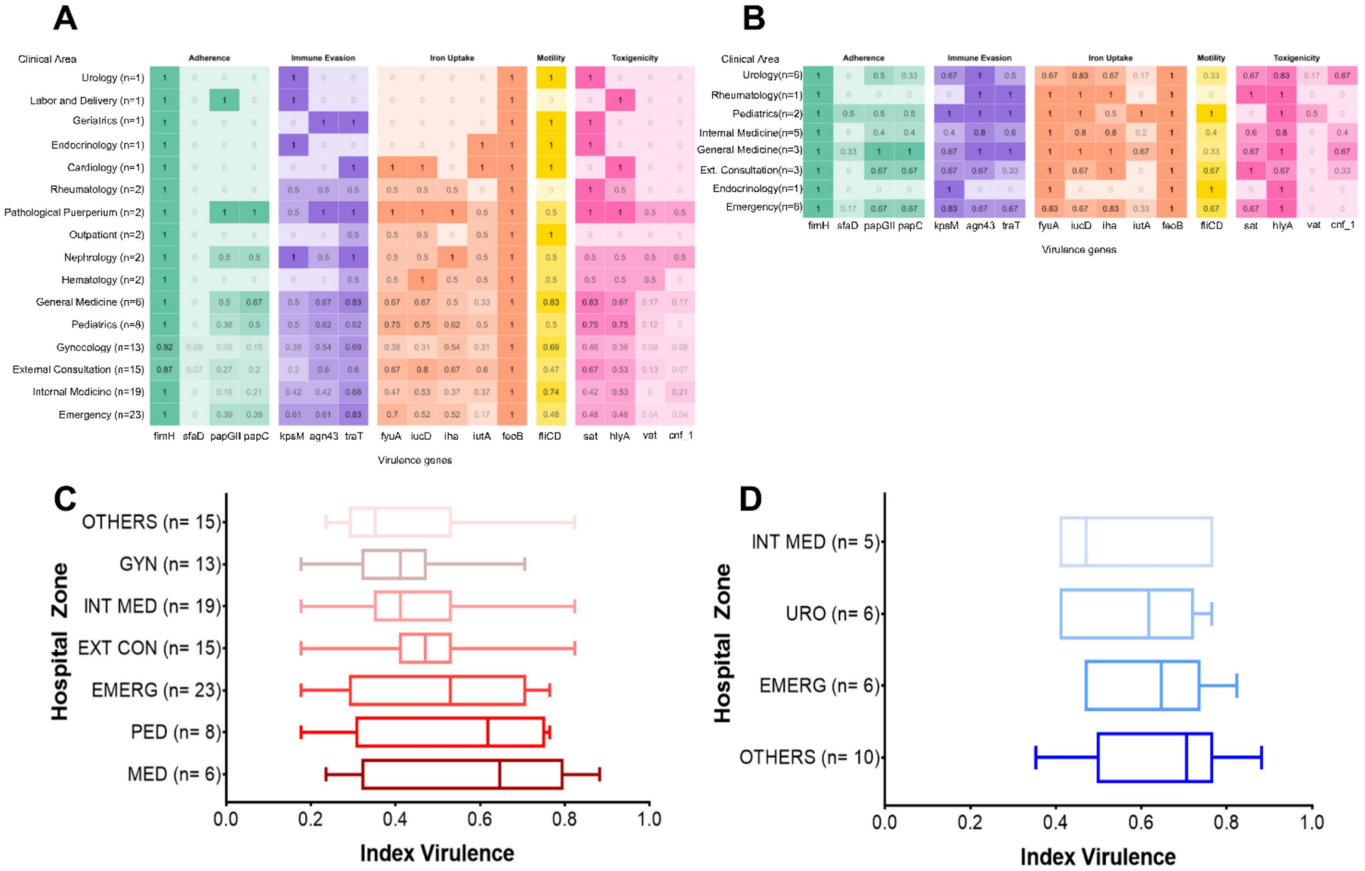
Distribution of virulence-associated genes (VAGs) by hospital ward. **(A)** Female patients; **(B)** male patients. Virulence indices by hospital ward in **(C)** female patients; **(D)** male patients. GYN, Gynecology; INT, Internal medicine; EXT CON, External consultation; EMERG, Emergency; PED, Pediatrics; MED, Medicine; URO, Urology. In the box plots, each box represents the interquartile range (IQR), i.e., the interval between the 25th percentile (Q1) and the 75th percentile (Q3), encompassing the central 50% of the data. The line within the box indicates the median, while the whiskers extend to the minimum and maximum values within 1.5 times the IQR.

The distribution of virulence genes was further analyzed by hospital ward of origin. Among isolates from female patients, the General Medicine, Gynecology, Outpatient Clinics, Internal Medicine, and Emergency wards showed a higher prevalence of each virulence gene (Figure 2A). However, these differences were not statistically significant (*p* > 0.05). Similarly, in male patients, a higher prevalence of virulence genes was noted in isolates from General Medicine, Urology, Pediatrics, Emergency, and Outpatient wards (Figure 2B), yet no statistically significant differences were detected (*p* > 0.05).

To provide a more integrated assessment of the virulence gene load per strain, individual virulence indices were calculated by dividing the number of detected genes by the total number evaluated (*n =* 17). These indices were compared across hospital wards using the non-parametric Kruskal–Wallis test, followed by Dunn’s *post hoc* test with Bonferroni correction. Boxplots were generated to visualize the distribution of virulence indices by ward for female (Figure 2C) and male (Figure 2D) patients. Each plot displays the median, interquartile range (IQR), and outliers, allowing for the evaluation of dispersion patterns and relative virulence burden across clinical units. To ensure robust statistical analysis, wards with fewer than five isolates were grouped under the category “Others.”

Among female patients, although no statistically significant differences were observed between wards (*p* > 0.05), notable trends were identified. Isolates from the Medicine, Pediatrics, and Outpatient wards showed proportionally higher virulence indices compared to those from Gynecology and Internal Medicine. However, no pairwise comparison reached statistical significance (*p* > 0.99) (Figure 2C). Similarly, in male patients, no statistically significant differences were observed (*p* > 0.05). Nonetheless, the highest virulence indices were found in isolates from wards classified under “Others” and from the Emergency ward, while the lowest indices were recorded in Urology and Internal Medicine (Figure 2D). Although these differences were not statistically significant, the observed trends may reflect a differential distribution of more virulent strains across specific clinical areas.

#### 3.2.3 Virulence profiles

The analysis of virulence profiles revealed high diversity, with a total of 92 distinct profiles distributed between male and female patients (Supplementary material 1A). Among female patients, 70 unique profiles were identified; of these, 14 were shared among 44 isolates (Supplementary material 1B). In male patients, 22 distinct profiles were detected, two of which were shared among seven isolates (Supplementary material 1C).

#### 3.2.4 Co-ocurrence of virulence genes

Several virulence genes were found to co-occur within the same clinical isolates. Statistical analysis was conducted to assess the significance of this co-occurrence (Supplementary material 1D). Significant co-occurrence was observed for multiple genes, particularly *agn43*, *cnf*-1, *fyuA*, *hlyA*, *iucD*, *kpsM*, *papC*, *papG*-II, *sat*, *sfaD*, *traT*, and *vat*, suggesting potential genetic linkage, likely mediated by mobile genetic elements.

### 3.3 Antimicrobial resistance

#### 3.3.1 Overall resistance by sex

Resistance to 17 antibiotics, including those recommended for UTI treatment in Honduras, was evaluated. Overall, the resistance index (*RI*) in isolates from female patients (*n =* 99) was 0.27 ± 0.20 (median: 0.2353), while isolates from male patients (*n =* 27) showed a significantly higher *RI*, with a mean of 0.46 ± 0.22 and a median of 0.5294. Since data from the female group did not follow a normal distribution (*p* < 0.0001; Shapiro–Wilk test), the nonparametric Mann–Whitney test was applied, revealing a statistically significant difference between groups (*p* < 0.0001). This result suggests a higher burden of antimicrobial resistance in isolates from male patients compared to those from female patients.

#### 3.3.2 Resistance by age group and multidrug resistance

Comparison of resistance indices by age group within each sex showed no statistically significant differences. However, males over 50 years of age exhibited significantly higher *RIs* than females under 5 years (*p* = 0.0108), females aged 13 to 50 years (*p* = 0.0077), and females over 50 years (*p* = 0.0410). These findings suggest a trend toward increased antimicrobial resistance in older male patients, although caution is warranted due to limited sample sizes in some subgroups, which may reduce statistical power and underestimate actual differences, particularly among males under 50.

Based on antibiotic resistance profiles, 67% of the 126 isolates were classified as MDR (multidrug-resistant), 2% as XDR (extensively drug-resistant), and 31% as NMDR (non-multidrug-resistant). The highest relative proportion of MDR isolates was observed in male patients (88%) compared to 63% in female patients. This difference was statistically significant (*p* = 0.009).

#### 3.3.3 Prevalence of antibiotic resistance

**Figure 3:**
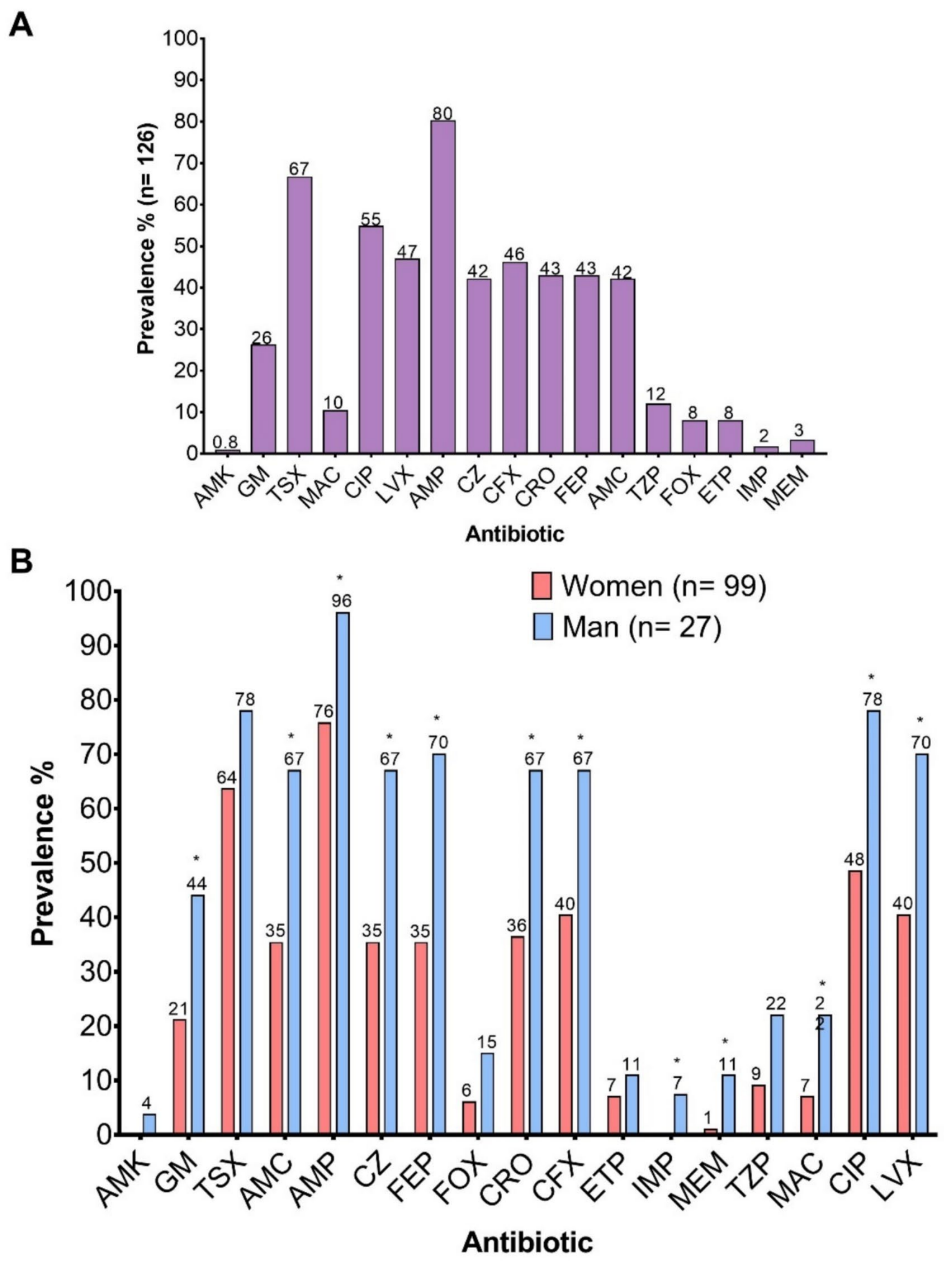
Prevalence of resistance to the 17 antibiotics evaluated. **(A)** Overall prevalence; **(B)** Prevalence according to the sex of the patients. AMK, Amikacin; GM, Gentamicin; TSX, Trimethoprim-Sulfamethoxazole; AMC, Amoxicillin-Clavulanic Acid; AMP, Ampicillin; CZ, Cefazolin; FEP, Cefepime; FOX, Cefoxitin; CRO, Ceftriaxone; CFX, Cefuroxime; ETP, Ertapenem; IMP, Imipenem; MEM, Meropenem; TZP, Piperacillin-Tazobactam; MAC, Nitrofurantoin; CIP, Ciprofloxacin; LVX, Levofloxacin.

The frequency of resistance to key antimicrobial categories included in the study was evaluated. Among the 126 clinical isolates analyzed, the highest prevalence of resistance was observed against penicillins (AMP), sulfonamides (TSX), fluoroquinolones (LVX, CIP), and second- to fourth-generation cephalosporins (CFX, CRO, FEP) (Figure 3A). Resistance levels were consistently higher in isolates from male patients compared to those from female patients across all tested antimicrobials. However, statistically significant differences were observed only for aminoglycosides (GM, *p* = 0.0246), penicillins (AMP, *p* = 0.0151), *β*-lactam/β-lactamase inhibitor combinations (AMC, *p* = 0.0044), first- to fourth-generation cephalosporins (CZ, *p* = 0.0044; CRO, *p* = 0.0078; FEP, *p* = 0.0018; CFX, *p* = 0.0177), carbapenems (IMP, *p* = 0.0446; MEM, *p* = 0.0307), nitrofurans (MAC, *p* = 0.0327), and fluoroquinolones (CIP, *p* = 0.0085; LVX, *p* = 0.0084) (Figure 3B).

The observed differences were supported by odds ratios (OR) and relative risk (RR) values indicating a higher probability of resistance in male patients, with confidence intervals that in all cases excluded the null value (Supplementary material 2A).

When comparing resistance by age and sex, adult males over 50 years of age exhibited the highest resistance burden, with rates exceeding 50% for several antibiotics, including gentamicin (GM), ampicillin (AMP), amoxicillin-clavulanic acid (AMC), cephalosporins, and fluoroquinolones. Elevated resistance levels were also observed in males aged 13–50 years, although slightly lower (Supplementary material 2A). In contrast, isolates from pediatric males showed low prevalence of resistance, with no cases reported for carbapenems and only one for fluoroquinolones. Among adult and pediatric females, resistance levels were moderate overall; however, in girls under 5 years and those aged 6–12, very high prevalence was observed, particularly for trimethoprim/sulfamethoxazole (up to 100%), amoxicillin-clavulanic acid (40%), and ampicillin (80%) (Supplementary material 2B).

#### 3.3.4 ESBL, carbapenemase production, and multidrug resistance

According to the Phoenix™ system results, 53 (42.06%) of the 126 isolates were extended-spectrum *β*-lactamase (ESBL) producers, while 10 (7.93%) produced carbapenemases. Sex-based analysis revealed that 37% of the isolates from female patients and 59% of those from male patients were ESBL producers, a statistically significant difference (*p* = 0.049). In contrast, carbapenemase production was similar in both groups, with 8% in females and 7% in males, showing no significant difference (*p* > 0.05). Notably, a considerable proportion of ESBL-producing strains were observed in female pediatric patients, but not in male infants (Supplementary material 2B).

#### 3.3.5 Co-ocurrence of antibiotic resistance

As multiple isolates showed simultaneous resistance to various antimicrobial classes, Pearson correlation analysis was conducted to identify significant statistical associations among resistance profiles, including phenotypic profiles of ESBL and carbapenemase (CAR) production.

This analysis revealed positive associations between multiple pairs of antibiotics, particularly a strong correlation between ceftriaxone (CRO) and ciprofloxacin (CIP) (r = 0.433, *p* < 0.001), suggesting concurrent resistance to β-lactams and fluoroquinolones. High correlations were also found between CRO and other cephalosporins such as cefepime (FEP) (r = 0.87, *p* < 0.001) and cefuroxime (CFX) (r = 0.873, *p* < 0.001), as well as with ertapenem (ETP) (r = 0.28, *p* < 0.001) and meropenem (MEM) (r = 0.209, *p* = 0.009). CIP also showed significant correlations with GM, AMC, AMP, FEP, and nitrofurantoin (MAC), among others (r values between 0.20 and 0.43; *p* < 0.01) (Supplementary material 2C).

The Pearson correlation analysis also demonstrated a highly significant association between the ESBL phenotype and resistance to cephalosporins such as CRO (r = 0.886), FEP (r = 0.821), and CFX (r = 0.794), all with *p* < 0.001. Positive correlations were also found between ESBL and resistance to fluoroquinolones such as CIP (r = 0.355, *p* < 0.001), as well as piperacillin-tazobactam (TZP) (r = 0.282) and MAC (r = 0.187). Regarding the CAR phenotype, strong correlations were identified with ETP (r = 0.891), imipenem (IMP) (r = 0.198), and MEM (r = 0.449), all statistically significant (*p* < 0.05).

#### 3.3.6 Virulence vs. resistance

Additionally, when comparing the virulence and resistance indices of the 126 isolates, a weak but statistically significant positive correlation was observed between the two variables (r = 0.1796, *p* = 0.0442). This finding suggests that some isolates simultaneously carry a high virulence gene load and an extensive antimicrobial resistance profile.

### 3.4 Phylogenetic groups

#### 3.4.1 Phylogroups in the general population

**Figure 4:**
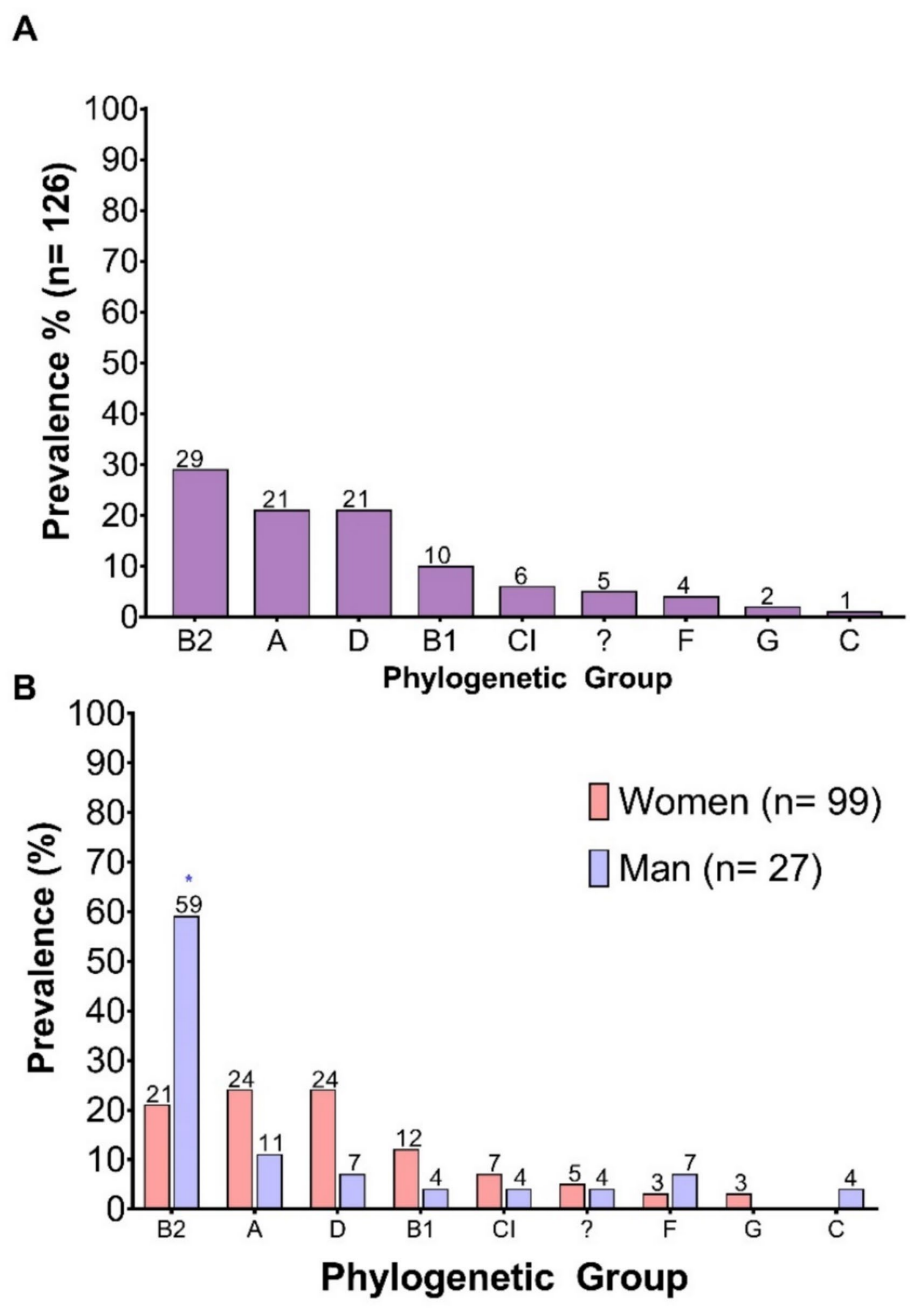
Prevalence of *E. coli* phylogenetic groups. **(A)** Prevalence of each phylogenetic group among the total number of clinical isolates analyzed; **(B)** Prevalence according to the sex of the patient. “?”: Unknown phylogroup.

The phylogenetic groups were identified, revealing a diverse distribution among the 126 strains evaluated. The most prevalent phylogroup was B2, present in 29% of the isolates, followed by groups A and D, both with a prevalence of 21%. Lower proportions were observed for phylogroups B1 (10%), Clade I (6%), F (4%), G (2%), and C (1%). Additionally, 5% of the isolates could not be assigned to any known phylogroup and were classified as “Unknown Phylogroup” (Figure 4A).

#### 3.4.2 Phylogroups by sex

When analyzing distribution by sex, a greater phylogenetic diversity was observed in isolates from female patients compared to those from males. In female samples, the most represented phylogroups were A and D, each with a prevalence of 24%, followed by B2 at 21%. In contrast, male isolates showed a marked predominance of phylogroup B2, accounting for 59%, a statistically significant difference compared to females. Additionally, phylogroup C was exclusively identified in isolates from male patients (Figure 4B). The higher prevalence of phylogroup B2 among isolates from male patients aligns with the increased virulence gene burden observed in this group.

#### 3.4.3 Phylogroups by hospital ward

**Figure 5:**
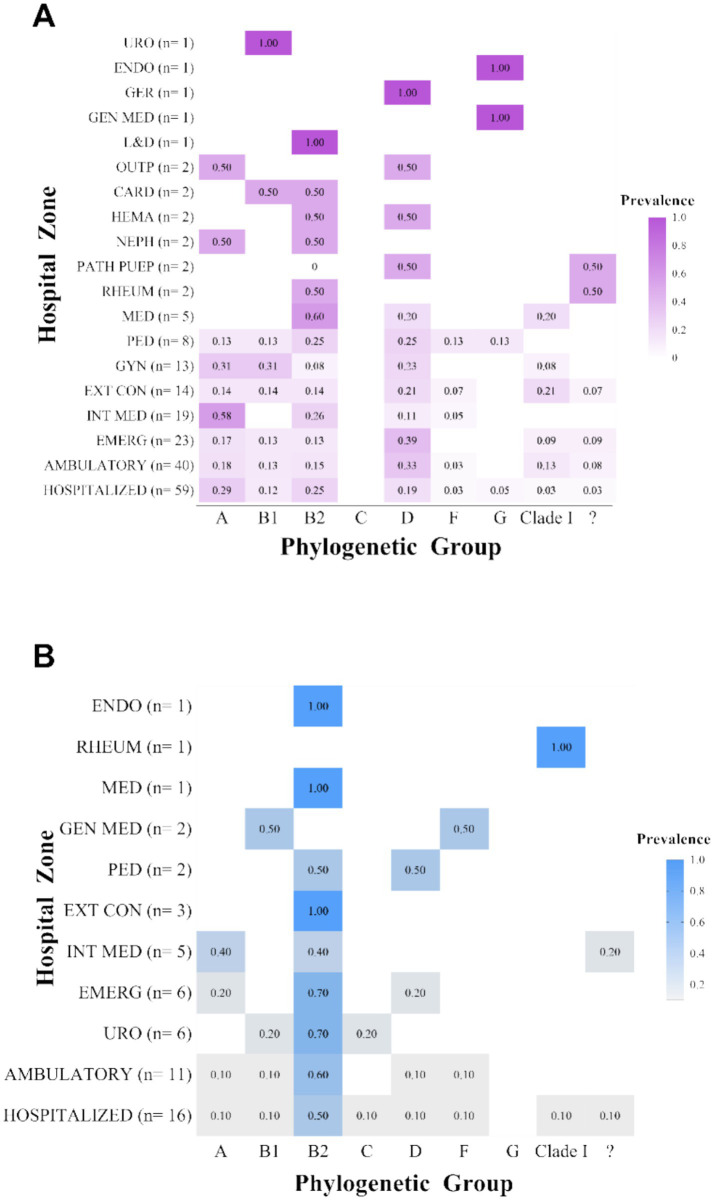
Distribution of phylogenetic groups by hospital ward. **(A)** Distribution of phylogroups by hospital ward in female patients; **(B)** Distribution of phylogroups by hospital ward in male patients. “?”: Unknown phylogroup.

After characterizing the overall prevalence of phylogroups among the clinical isolates, their distribution across different hospital wards was explored in order to identify potential associations with the clinical environment of origin. To this end, isolates were stratified according to the patient’s sex and the hospital ward of origin, and the relative proportions of each phylogroup within each group were calculated (Figures 5A,B). Although apparent differences in distribution were observed, no statistically significant associations were found between phylogenetic groups and hospital areas (*p* > 0.05).

#### 3.4.4 Virulence and phylogenetic groups

Differences in the total number of virulence genes among the various phylogenetic groups were assessed using the virulence index (VI). Due to the small sample sizes of phylogroups C (*n =* 1) and G (*n =* 3), these were grouped under the category “Others” to avoid statistical bias.

**Figure 6:**
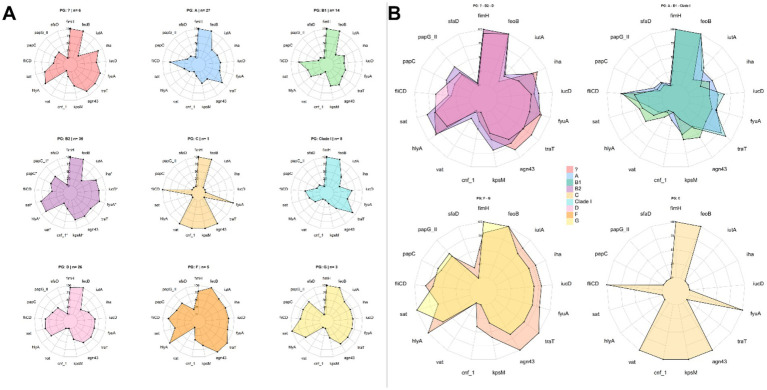
Virulence and phylogenetic groups. **(A)** Distribution of virulence genes by phylogenetic group; **(B)** Phylogroups showing similarities in the distribution of virulence genes. The presence of asterisks indicates a statistically significant association (*p* < 0.05).

The analysis revealed statistically significant differences among phylogroups (*p* < 0.05). In particular, phylogroup B2 showed a significantly higher VI compared to phylogroups A (*p* = 0.0495) and B1 (*p* = 0.0406), reinforcing its association with a higher genetic virulence load. While no significant differences were observed with other groups, it is noteworthy that phylogroup F displayed an even higher mean than B2. However, this difference was not statistically significant, likely due to the small sample size of group F (*n =* 5), which limits the statistical power of the analysis. Similarly, other groups with low representation, such as Clade I or those classified as “Others,” showed variable medians without reaching statistical significance (Supplementary Figure 2). These findings suggest that, although phylogroup B2 harbors the highest proportion of strains with multiple virulence genes, the pathogenic potential of other phylogroups, such as F, may be underestimated and should be further assessed in studies with larger sample sizes. When examining the distribution of virulence genes by phylogenetic group, similar patterns were observed among several phylogroups, particularly B2, D, and those classified as unknown (Figure 6B). This trend was consistent in isolates from female patients but was not observed in those from male patients (Supplementary Figures 3, 4, respectively).

Additionally, an analysis was conducted to determine whether specific virulence genes were more prevalent in a given phylogenetic group. Overall, phylogroup B2 displayed the highest number of statistically significant associations, with a high prevalence of virulence genes. Notably, positive associations were found for the genes *papG-II*, *papC*, *sat*, *hlyA*, *vat*, *cnf-1*, *kpsM*, *fyuA*, *iucD*, and *iha*, all of which were detected more frequently in isolates belonging to this phylogroup (Figure 6A). Odds ratio (OR) and relative risk (RR) values (Supplementary material 3) confirmed these associations, indicating that B2 strains not only exhibited a higher prevalence of these genes but also had a significantly greater likelihood of harboring them compared to other phylogroups. Some genes, such as *sat* (OR = 4.52; RR = 1.68), *cnf-1* (OR = 4.51; RR = 3.43), *vat* (OR = 4.3; RR = 3.75), *hlyA* (OR = 3.96; RR = 1.57), and *kpsM* (OR = 3.25; RR = 1.68), showed the highest values, indicating a strong association with key properties such as cytotoxicity and immune evasion.

In contrast, phylogroups A and B1 showed a lower prevalence of most of the aforementioned virulence genes, with statistically significant differences. These findings support the association of phylogroup B2 with virulence factors related to cytotoxicity and adhesion at both bladder and renal levels, and are consistent with previous evidence describing phylogroups A and B1 as lower-virulence or commensal groups.

#### 3.4.5 Resistance and phylogenetic group

**Figure 7:**
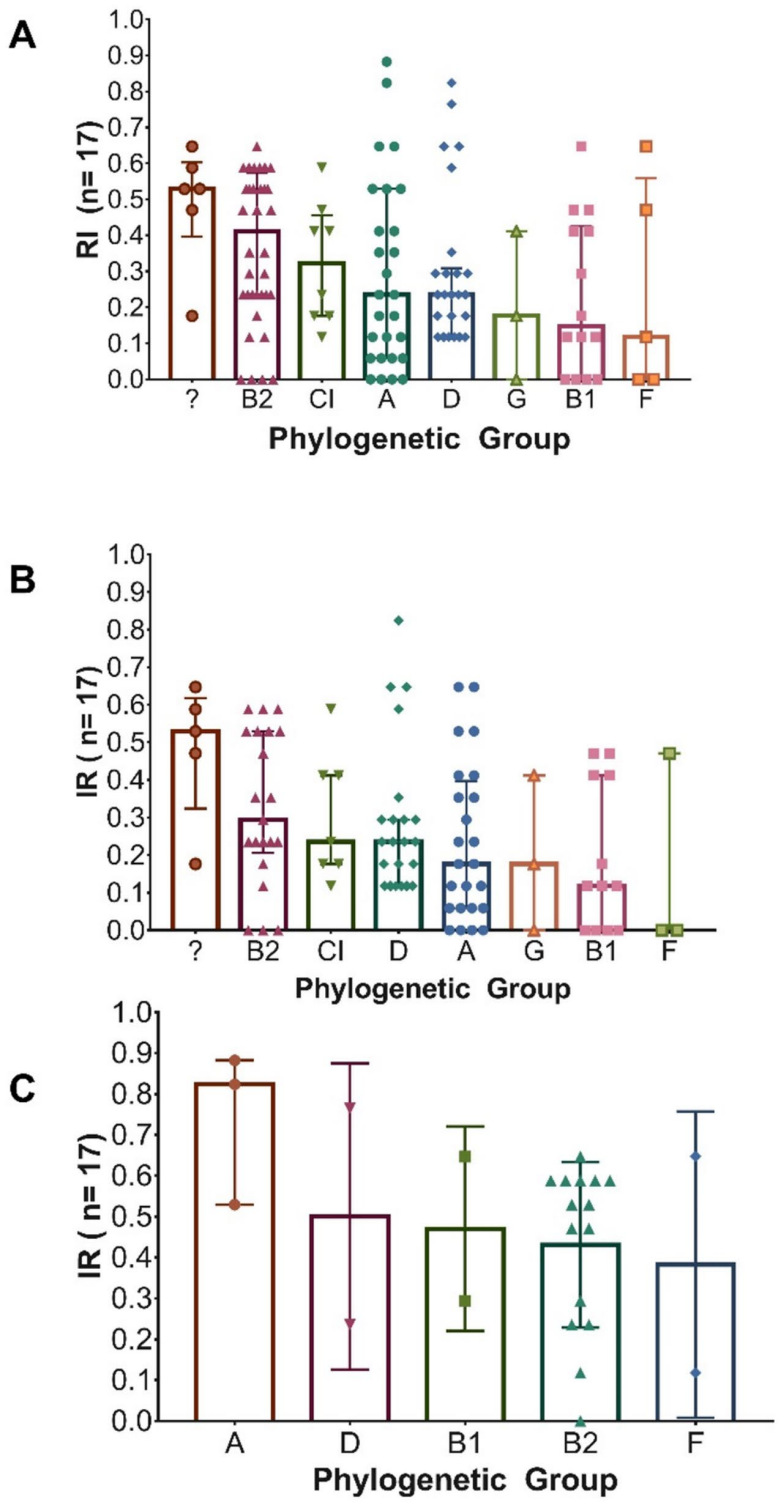
Distribution of the total number of antibiotics to which *Escherichia coli* isolates are resistant, according to their phylogenetic group. **(A)** All isolates (*n =* 126); **(B)** Isolates from female patients (*n =* 99); **(C)** Isolates from male patients (*n =* 27). In each graph, bars indicate the median and interquartile ranges (IQR).

Differences in the resistance index (*RI*) among the various phylogenetic groups were evaluated using the non-parametric Kruskal–Wallis test, as the assumption of normality was not met. Although visual differences were observed in the range of RI values for certain phylogroups—particularly for group A (in males), B2 (in all isolates), and the unknown group (in females)—the analyses conducted on the overall population, as well as those stratified by sex, did not show statistically significant differences (*p* = 0.176) (Figures 7A–C). These findings suggest that, based on the available data, no systematic difference in resistance index can be confirmed among the phylogenetic groups evaluated.

When evaluating the association between resistance to specific antibiotics and phylogenetic groups, statistically significant differences (*p* < 0.05) were observed in certain cases. Among the isolates obtained from male patients, a higher prevalence of resistance to cefoxitin (FOX), ertapenem (ETP), and imipenem (IMP) was recorded, each with a 67% prevalence in phylogenetic group A, compared to other groups. In female patients, a greater number of carbapenemase-producing isolates (62.5%) was identified in group A, as well as higher resistance to gentamicin (GM) in group B2 (57%), to ampicillin (AMP) in group D (96%), and to levofloxacin (LVX) and ciprofloxacin (CIP) in the undetermined phylogenetic group (100%). All these associations were statistically significant (*p* < 0.05).

### 3.5 Eric PCR

To evaluate the potential clonal relationship among clinical uropathogenic *E. coli* isolates, a clustering analysis was performed based on profiles obtained by ERIC-PCR. This analysis was organized at three levels: by hospital ward, among non-hospitalized patients, and among hospitalized patients. At the hospital ward level, possible clonal relationships were identified among isolates from eight clinical departments. The wards with the highest number of clusters were Emergency (5 clusters; 13 strains), Internal Medicine (7 clusters; 14 strains), Gynecology (2 clusters; 6 strains), General Medicine (2 clusters; 4 strains), Pathological Puerperium (1 cluster; 2 strains), Endocrinology (1 cluster; 2 strains), Pediatrics (1 cluster; 2 strains), Urology (2 clusters; 4 strains), and Outpatients (1 cluster; 2 strains) (Supplementary Figures 5A–P). Among hospitalized patients, 58 isolates were grouped into 20 clusters (Supplementary Figures O,P), while in non-hospitalized patients, 39 isolates were grouped into 16 clusters (Supplementary Figure 5P).

**Figure 8:**
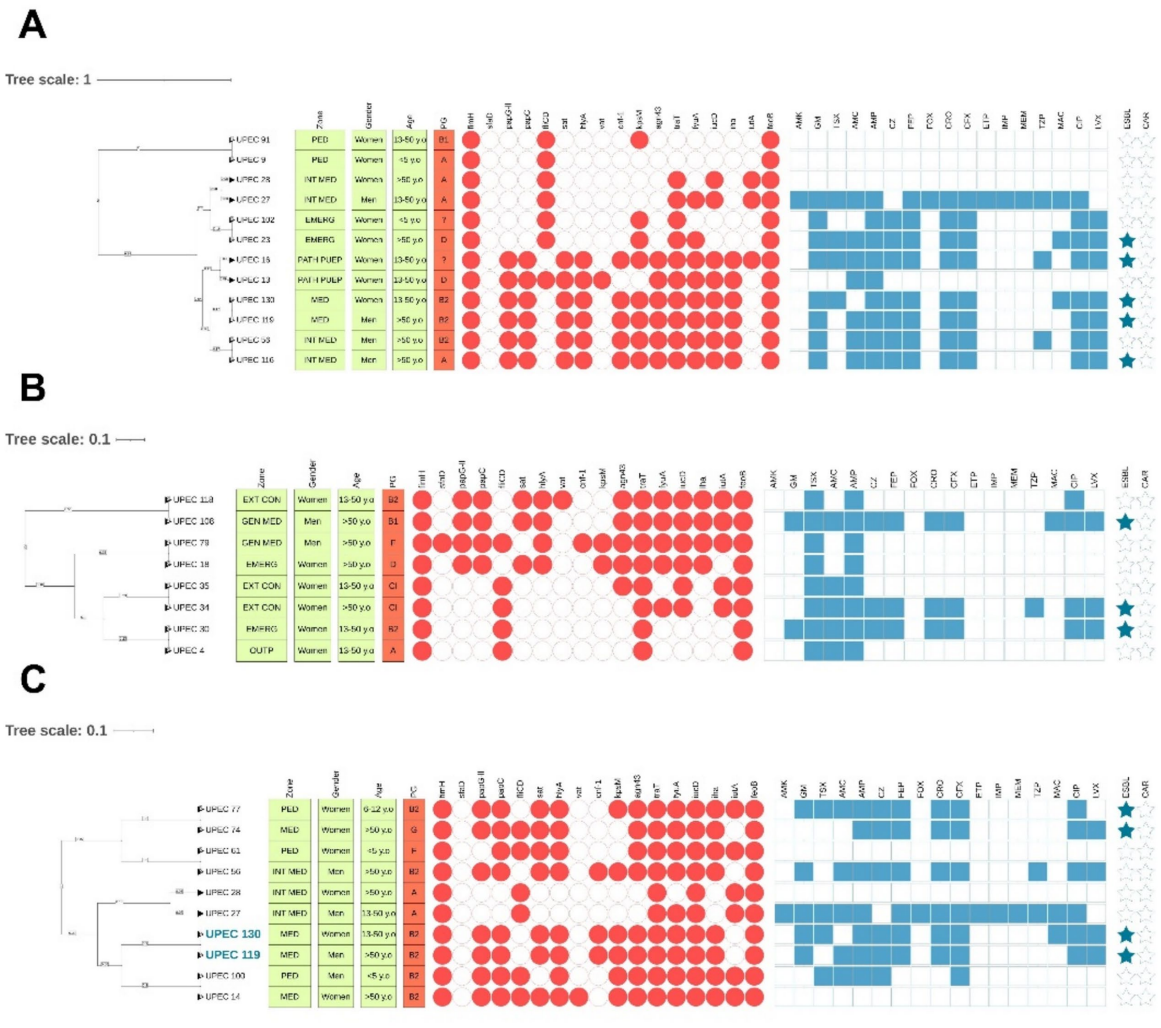
Clinical isolates with the highest similarity, according to clustering criteria, virulence profiles, and antibiotic resistance phenotypes. **(A)** Analysis by hospital ward; **(B)** Analysis in hospitalized patients; **(C)** Analysis in outpatients. Isolates shown in blue exhibit the highest similarity in virulence, resistance, and phylogenetic groups.

Despite the high similarity in banding patterns obtained through ERIC-PCR, we observed variations in resistance and virulence profiles, and even in phylogenetic grouping. In this context, it is important to consider that ERIC sequences do not differentiate specific virulence or antibiotic resistance genes, which can be acquired through horizontal gene transfer events. Similarly, phylogenetic groups are determined by specific genes that cannot be distinguished via ERIC-PCR. However, we identified some isolates with features suggestive of possible clonality: they showed identical ERIC profiles according to our cut-off criteria, along with high similarity in both virulence and antibiotic resistance profiles. These isolates were detected by hospital ward (Figure 8A), in the group of hospitalized patients (Figure 8B), and the outpatient group (Figure 8C). Figure 8 visually summarizes some of the clusters with the highest similarity, integrating genomic, phenotypic, and epidemiological profiles.

## 4 Discussion

Urinary tract infections (UTIs) pose a significant public health challenge due to their high incidence and the growing prevalence of multidrug-resistant bacterial strains (Magiorakos et al., 2012; Flores-Mireles et al., 2015). Uropathogenic *Escherichia coli* (UPEC) is the main causative agent, noted for its high capacity to acquire both virulence and antibiotic resistance genes (Ballesteros-Monrreal et al., 2021; Akhand and Karicheri, 2022). In Honduras, information on UPEC is scarce; therefore, characterizing local strains is essential to improve microbiological diagnosis, empirical treatment, and epidemiological surveillance. In this study, 126 clinical isolates confirmed as *E. coli* were analyzed, of which 99 (78.6%) were obtained from women and 27 (21.4%) from men. This higher proportion in women aligns with the literature, which points to anatomical and hormonal factors as predisposing elements for UTIs (Flores-Mireles et al., 2015; Raphael et al., 2024). These factors include the shorter female urethra compared to that of males, as well as the proximity of the anus and vagina, which facilitates ascending colonization (He et al., 2025; Deltourbe et al., 2022).

One of the findings in this study was that most isolates came from patients aged 13 to 50, followed by patients over 50, with no significant differences between sexes. In the 13–50 age group, events such as sexual activity, use of contraceptives, pregnancy, and in some cases prostatic hyperplasia occur more frequently—factors that are all associated with a higher risk of UTI (He et al., 2025; Deltourbe et al., 2022). Although no detailed clinical information was collected from patients in this study, these factors have been widely described as risk elements for disease development. Therefore, their influence cannot be ruled out and should be considered in future research that integrates clinical, microbiological, and demographic variables more comprehensively.

### 4.1 Virulence determinants

On the other hand, the analysis of virulence genes revealed significant differences between clinical isolates from male and female patients. Overall, strains from male patients exhibited a significantly higher virulence gene burden, with an average of 10.48 genes per strain, compared to 8.06 genes in female patients (*p* = 0.0029). This difference suggests that infectious episodes in male patients may be associated with strains possessing a greater pathogenic potential, which aligns with previous studies that have described a higher requirement for adaptability to colonize the male urinary tract, particularly in the presence of obstructive factors or urinary catheterization (Ruiz et al., 2002; AP et al., 2017).

Regarding age group distribution, results in females showed a higher mean number of genes in patients under 5 years old; however, this difference did not reach statistical significance. In males, the 6-to-12-year-old group stood out with a significantly higher genetic load than that observed in adolescents and adults (*p* < 0.05). This finding suggests that, in specific pediatric subgroups, pathogenesis may involve a higher number of virulence factors, which could translate into more aggressive clinical presentations or increased risk of recurrence. The elevated virulence gene load in isolates from pediatric patients has been previously reported by other authors (Hernández-Chiñas et al., 2019; Luna-Pineda et al., 2018), and although the sample size in our study limits definitive conclusions, these data suggest that the pediatric population, particularly school-aged boys, represents a risk group that warrants closer clinical and microbiological monitoring. Implementing follow-up strategies in this cohort could be key to detecting emerging virulence patterns, preventing complications, and timely adjusting therapeutic decisions.

Among the most prevalent genes, *fimH* (96%) and *feoB* (100%) were identified, both essential for initial adherence to the uroepithelium and iron acquisition, respectively, as well as for urinary tract colonization, particularly in nutrient-limited environments (Alam Parvez and Rahman, 2018). The high frequency of these genes is consistent with their central role in the pathophysiology of UPEC and has been widely reported (Ballesteros-Monrreal et al., 2021; Zhao et al., 2009; Yun et al., 2014; Tabasi et al., 2015; Sarshar et al., 2020). However, when stratified by sex, significant differences were observed in the prevalence of genes with more specialized functions. In males, there was a higher presence of genes related to renal-level adherence (*papG*-II, *papC*), toxins (*hlyA*, *cnf*-1), capsule formation (*kpsM*), and iron acquisition systems (*fyuA*, *iucD*, *iha*), all of which are associated with increased persistence and tissue damage in complicated infections (Ballesteros-Monrreal et al., 2021).

These results are consistent with previous reports: Kudinha et al. (2013) described a virulence gradient with higher prevalence of virulence factors in pyelonephritis compared to cystitis and healthy controls; Firoozeh et al. (2014) showed that pyelonephritis isolates harbor a greater diversity of virulence genes, particularly pap, traT, aer, hly, and PAIs; Hyun et al. (2022) identified sex-based differences with male isolates enriched in toxin genes such as hlyA and cnf1; and Hsiao et al. (2024) demonstrated that phylogroup B2 predominates in UTI and bacteremic cases, with genes such as papGII enriched in invasive infections (Hyun et al., 2022; Hsiao et al., 2024; Firoozeh et al., 2014; Kudinha et al., 2013). Collectively, these findings support our observation that isolates from male patients harbor more virulence determinants and are also associated with complicated clinical outcomes.

Regarding the virulence profiles of the isolates, high variability was observed, with a total of 92 distinct profiles: 14 were shared among multiple isolates from female patients, and only 2 among male isolates. Notably, isolates with shared profiles were not concentrated in a specific hospital ward or age group, suggesting a random and unfocused distribution of genetically similar strains within the hospital environment, with no clear evidence of clonal clustering within this dataset. Additionally, the low frequency of shared profiles is consistent with the high heterogeneity observed in the ERIC-PCR patterns, suggesting that most cases correspond to independent infectious events and a high diversity in infection sources. Nevertheless, ERIC-PCR results revealed two isolates that showed high similarity in their virulence characteristics, resistance, phylogenetic group, and hospital origin (Figure 8C). This finding underscores the importance of implementing continuous molecular surveillance to identify potential outbreaks, common transmission routes, or the emergence of successful clones with predominant virulence profiles.

Likewise, the high heterogeneity observed in virulence profiles may be related to the acquisition and maintenance of genes through horizontal transfer mechanisms, such as plasmids and pathogenicity islands (PAIs), reflecting the remarkable adaptability of uropathogenic isolates to diverse ecological niches and selective pressures. In our analysis, the significant co-occurrence of genes such as *papG*-II, *papC*, *sfaD*/*focC*, *vat*, *fyuA*, *kpsM*, and *iucD* is consistent with the findings of Ballesteros-Monrreal et al. (2021), who identified similar clusters in uropathogenic *E. coli* strains. More recently, Ballesteros-Monrreal et al. (2024) corroborated the presence of these PAIs through molecular characterization, suggesting that the observed gene clustering in our isolates likely reflects the functional and structural organization of these genetic elements rather than random distribution. This finding supports the hypothesis that the simultaneous presence of multiple virulence factors may be mediated by shared genomic structures. Moreover, considering that genes such as *iutA* and *iucD* have previously been associated with ColV-type plasmids (Johnson et al., 2006; Johnson and Nolan, 2009), it is reasonable to assume that some of our isolates may carry plasmids with horizontal dissemination capacity. This aspect is particularly relevant in hospital settings, where the circulation of strains harboring PAIs and plasmids contributes not only to pathogenic potential but also to the spread of antimicrobial resistance. Future studies should aim to confirm the presence and characterize the structure of such plasmids to better understand their role in the dissemination of resistance and virulence determinants. Ideally through whole-genome sequencing (WGS) to resolve their genetic context and evolutionary dynamics.

### 4.2 Antimicrobial resistance

Regarding the characterization of the isolates based on antibiotic resistance, the analysis of the resistance index (*RI*) revealed significant differences between clinical isolates according to the patient’s sex. Isolates from male patients exhibited a significantly higher antimicrobial resistance burden, with an average *RI* of 0.46, compared to 0.27 in female-derived isolates (*p* < 0.0001). Additionally, 69% of all isolates were classified as MDR (including XDR), with the highest proportion of MDR found in male patients (88%; *p* = 0.009). This difference reinforces the previously observed trend in virulence gene burden, suggesting that strains isolated from male patients not only possess greater pathogenic potential but also exhibit increased antimicrobial resistance. However, one of the limitations of our study is the smaller male sample size (*n =* 27), which restricts the possibility of drawing definitive conclusions in this subgroup, although the observed trends are consistent with international evidence (Ruiz et al., 2002; AP et al., 2017; Khanal et al., 2024).

These results are consistent with findings by Khanal et al. (2024), who analyzed over 85,000 uropathogenic *E. coli* isolates in Australia and found a high prevalence of MDR strains in both hospital and community settings, with a significantly higher proportion in males compared to females (16.5% vs. 12.8% in hospitals; 6.4% vs. 5.2% in the community; *p* < 0.001). Although the Australian study also lacked individual clinical metadata, the authors attributed these differences to the fact that urinary tract infections in males are more frequently associated with conditions that complicate the clinical course, such as benign prostatic hyperplasia, urolithiasis, use of urinary catheters, history of urological instrumentation, or prior surgical interventions (Khanal et al., 2024). These conditions lead to urinary flow obstruction, increased risk of persistent colonization, and the need for prolonged or broad-spectrum antibiotic treatments, favoring the selection of resistant strains. Similar to our findings, Khanal et al. (2024) noted that, despite UTIs being more frequent in females, the strains isolated from males showed higher multidrug resistance rates (Khanal et al., 2024). This disparity suggests that UTIs in males may be caused by strains with more complex virulence and resistance profiles. This finding underscores the need to adapt empirical treatments not only to the type of infection but also to the epidemiological features associated with patient sex.

Among the 17 antibiotics tested, the highest prevalence of resistance in the 126 strains was observed for AMP (80%), TSX (67%), fluoroquinolones (CIP and LVX), and several second- to fourth-generation cephalosporins. When stratified by sex, isolates from male patients showed higher resistance rates for all evaluated antibiotics, with statistically significant differences observed for aminoglycosides, simple and combination penicillins, cephalosporins, carbapenems, nitrofurans, and fluoroquinolones. Similarly, the ESBL phenotype was more prevalent in this group (59%; *p* = 0.049). These results are particularly relevant when contrasted with currently recommended empirical treatment regimens in Honduras (Honduras, 2019; Ucros, 2009; Giovanni Erazo Trimarchi et al., 2015; Honduras, 2016). For outpatient pediatric patients, first-line treatment includes TSX and AMC, whereas in hospital settings, AMP, GM, AMK, CRO, or ampicillin-sulbactam (AMS) are commonly used (Johnson et al., 2006; Johnson and Nolan, 2009; Ucros, 2009). However, our findings reveal a high prevalence of resistance to TSX (60–100%), AMP (83–85%), and AMC (27–29%) in pediatric isolates of both sexes (with resistance reaching 66.7% in males and 40% in females), as well as to GM (33.3%) in males and CRO (33.3%) in females (Supplementary material 2B), suggesting potentially limited efficacy of these therapeutic options in the pediatric population. This situation is particularly concerning in children under 5 years of age, not only due to the frequent use of these antibiotics as first-line treatments but also due to the detection of a high burden of virulence genes. The coexistence of these factors may compromise therapeutic success and worsen clinical prognosis. Additionally, among pediatric female patients, ESBL- and CAR-producing isolates were observed, whereas none were detected in male children (Supplementary material 2B). This difference may be explained by the higher frequency of UTIs in females, which increases exposure to antibiotic treatments and consequently the selective pressure favoring the emergence of MDR strains.

In adult outpatients, commonly used antibiotics include AMC, TSX, CIP, LVX, and CRO; in hospitalized patients, fluoroquinolones, CRO, AMS, TZP, and carbapenems such as ertapenem (ETP) are used (Giovanni Erazo Trimarchi et al., 2015; Loscalzo et al., 2022). In our study, high resistance prevalence was observed among adult patients against these antimicrobials, particularly TSX (60–83.3%), CIP (39–93.75%), and CRO (31–83%) (Supplementary material 2B). This resistance pattern is not unique to our setting: studies conducted in the USA and Australia have reported elevated resistance rates to these drugs, especially TSX, more commonly observed in patients with recurrent UTIs, anatomical abnormalities of the urinary tract, or recent antibiotic treatment history, which has raised concerns regarding its use as a first-line empirical therapy (Khanal et al., 2024; Wesolek et al., 2022). These findings also align with a systematic review and meta-analysis conducted in Mexico, which reported high resistance to ampicillin (79%), third-generation cephalosporins (40–50%), quinolones and fluoroquinolones (45–61%), as well as TSX (61.9%), along with a considerable prevalence of ESBL-producing isolates (39.46%) (Ballesteros-Monrreal et al., 2023). This body of evidence underscores the urgent need to reassess empirical treatment guidelines for UTIs, considering both selective pressure and local resistance contexts, which may vary significantly depending on geographic region.

The high prevalence of resistance to these antibiotics may be linked to well-established genetic mechanisms. For TSX, resistance has been associated with *dfrA* and *sul* genes, commonly found in mobile integrons (Poey et al., 2024). In the case of CIP, resistance can result from chromosomal mutations in *gyrA* and *parC*, as well as plasmid-mediated mechanisms involving efflux pumps (*qepA*), target protection (*qnrB*, *qnrA*, *qnrS*), or enzymatic modification [*aac(6′)-Ib-cr*] (Malekzadegan et al., 2019). Regarding CRO, one of the most frequent mechanisms is the production of extended-spectrum *β*-lactamases (ESBLs), which confer resistance to multiple cephalosporins. Notably, many of these determinants can coexist within conjugative plasmids that harbor resistance genes for various antimicrobial classes (Salah et al., 2019; Juraschek et al., 2022), facilitating co-selection phenomena.

The co-occurrence of antimicrobial resistance phenotypes in our isolates is unlikely to be random, but rather reflects their organization within mobile genetic elements such as integrons and conjugative plasmids, which frequently harbor multiple resistance determinants within the same replicon. This genetic architecture facilitates horizontal gene transfer and promotes co-selection, whereby exposure to a single antibiotic may indirectly maintain resistance to several unrelated drugs. Similar patterns have been described in Enterobacteriaceae worldwide, underscoring the role of these mobile elements in the persistence and dissemination of multidrug resistance. Future studies using whole-genome sequencing (WGS) will be essential to characterize the specific plasmids and integrons circulating in Honduras and to resolve the genetic context underlying these associations.

Although the presence of these genes was not directly assessed, a strong correlation was observed between resistance to ceftriaxone and ciprofloxacin (r = 0.433; *p* < 0.001), suggesting possible co-transmission of these phenotypes. Additionally, significant associations were found between the ESBL phenotype and resistance to both cephalosporins and fluoroquinolones (r = 0.355; *p* < 0.001), potentially indicating the presence of mobile genetic elements that integrate and disseminate gene clusters associated with multidrug resistance in the studied population, posing a considerable therapeutic threat, particularly in settings where these antimicrobials are frequently used empirically.

This pattern of co-dissemination of resistance mechanisms has been previously documented in recent studies conducted in Mexico. In clinical isolates from women with UTIs, Ballesteros-Monrreal et al. (2024) reported a high prevalence of *bla*_CTX-M*-2*_ and *aac(6′)-Ib* genes in isolates with simultaneous resistance phenotypes to *β*-lactams, fluoroquinolones, aminoglycosides, and ESBL production. Similarly, Marquez-Salazar et al. (2025) found that more than 95% of community-acquired *E. coli* isolates resistant to both β-lactams and fluoroquinolones carried ESBL and plasmid-mediated quinolone resistance (PMQR) genes—mainly *aac(6′)-Ib-cr*, *qnrB*, and *qepA*—pointing to the presence of conjugative plasmids carrying multiple resistance genes. The coexistence of these phenotypes may offer clues regarding potential resistance genotypes.

A limitation of our study is that we did not specifically detect these genetic determinants, highlighting the need for future investigations aimed at identifying genes associated with mobile genetic elements, as well as the circulating plasmids and integrons in *E. coli* strains from Honduras. Such research is essential to anticipate therapeutic efficacy crises and guide rational antibiotic use policies. Collectively, the findings of this study reveal a concerning landscape of antimicrobial resistance in uropathogenic *E. coli* strains in the Honduran context, necessitating a reevaluation of current therapeutic protocols and the strengthening of local surveillance. Integrating phenotypic resistance data with epidemiological and molecular information should be a priority for clinical decision-making and the formulation of antibiotic stewardship policies. In this regard, the use of personalized antibiograms, continuous training of medical personnel, and the incorporation of local data into clinical guidelines are key pillars for containing the spread of multidrug-resistant strains and preserving the effectiveness of available antimicrobials.

### 4.3 Phylogeny and clonality

The phylogenetic characterization of the 126 clinical *E. coli* isolates revealed a heterogeneous distribution, with a predominance of phylogroup B2 (29%), followed by A and D (21% each)—lineages commonly associated with extraintestinal infections. We also identified phylogroups B1, Clade I, F, G, and C, along with 5% of non-typeable strains. Notably, phylogroup G, recently incorporated into the scheme proposed by Clermont et al. (2019), was detected. Although rare, its clinical relevance cannot be ruled out due to its shared characteristics with B2. The application of this updated scheme may explain the lower proportion of non-typeable strains compared to previous studies. Nevertheless, the persistence of unclassified isolates suggests the possible presence of phylogenetic lineages not yet described in Honduras.

Regarding sex-specific distribution, greater phylogenetic diversity was observed among female patients, with predominance of groups A and D, whereas phylogroup B2 was significantly more prevalent in male patients (59%), potentially associated with a higher burden of virulence and resistance genes, as well as anatomical and clinical factors specific to males. Although no significant differences were found among hospital wards, we identified a trend toward the concentration of B2 strains in departments such as Internal Medicine and Urology, possibly reflecting their association with more complex clinical presentations. Virulence index analysis showed significantly higher values in phylogroup B2 compared to A and B1, with associations to key genes related to adhesion, cytotoxicity, immune evasion, and iron acquisition, such as *papG*-II, *papC*, *sat*, *hlyA*, *vat*, *cnf*-1, *kpsM*, *fyuA*, *iucD*, and *iha*. Although phylogroup F exhibited a high median index, the low frequency of isolates precludes definitive conclusions but suggests an underestimated pathogenic potential that warrants further investigation.

Regarding antimicrobial resistance, no significant differences were observed in the overall index across phylogroups, although sex-stratified patterns emerged: in males, phylogroup A showed higher resistance to carbapenems; in females, B2 exhibited elevated resistance to gentamicin, D to ampicillin, and non-typeable strains to fluoroquinolones. These findings point to the existence of phylogroup-specific profiles possibly modulated by distinct selective pressures. Our findings align with those of Ballesteros-Monrreal et al. (2021), who also reported high phylogenetic diversity in clinical isolates from women, with 25% of strains being non-typeable. In that study, phylogroup B2 harbored the highest virulence gene load (*iroN* 91%, *papC* 64%, *fyuA* 100%), whereas B1 harbored the greatest antimicrobial resistance. Similarly, Hemati et al. (2024) identified B2 as the most prevalent group (32%), followed by E, C, and B1, and reported that over half of the isolates could not be phylogenetically assigned. Kara et al. (2024) also highlighted B2 as the dominant phylogroup (45.5%), followed by E (22.4%) and 12% non-typeable strains. Complementarily, Wang et al. (2023) characterized 844 clinical isolates and confirmed the predominance of phylogroup B2 (64.8%), with the highest average number of virulence genes (10.1 per isolate). Moreover, although phylogroups C, E, and F were less frequent, they displayed high multidrug resistance rates, supporting the importance of monitoring minority lineages that may be acquiring clinically relevant traits.

### 4.4 Implications and future directions

Collectively, our data identify phylogroup B2 as the predominant uropathogenic lineage in this cohort, supported by its association with multiple virulence genes and prominent role in resistance profiles. These findings highlight the need to strengthen molecular surveillance of uropathogenic *E. coli* in response to increasing genetic diversity and the potential emergence of clinically significant new lineages. This study presents one of the first comprehensive molecular and phenotypic characterizations of UPEC in Honduras. Our findings demonstrate a high prevalence of virulence factors, MDR, and ESBL production among isolates from a tertiary-care hospital, with a particular impact on pediatric and male patient populations. The observed resistance to commonly prescribed antibiotics, including ampicillin, trimethoprim-sulfamethoxazole, fluoroquinolones, and cephalosporins, raises significant concerns regarding the continued effectiveness of empiric treatment protocols recommended in national guidelines.

Phylogenetic analysis revealed substantial genetic diversity, with phylogroup B2 emerging as the predominant lineage, associated with increased virulence and resistance. The ERIC-PCR profiles, in combination with overlapping resistance and virulence patterns, suggest potential clonal relationships and highlight the urgent need for enhanced molecular surveillance strategies. In a context where data on urinary tract infections remain limited, our results provide critical evidence to guide clinical decision-making, support antimicrobial stewardship initiatives, and reinforce microbiological surveillance at the local level.

Another limitation of our study is the short sampling period (2 months), which restricts epidemiological generalizability. Future investigations should extend the collection period to capture seasonal variation and temporal dynamics in UPEC populations. Recognizing this limitation underscores the importance of complementing cross-sectional molecular studies with longitudinal surveillance to fully appreciate the epidemiological behavior of uropathogenic *E. coli* in our setting.

Finally, one of the future goals of our research is the complete sequencing of the genomes of isolates exhibiting co-occurrence of virulence and resistance genes. Whole-genome sequencing (WGS) represents a more robust approach compared to PCR-based detection, as it allows for the comprehensive reconstruction of the genomic context in which these determinants are embedded. Through WGS, it will be possible to confirm the presence of mobilizable genetic elements such as conjugative plasmids, transposons, and pathogenicity islands, and to elucidate their structural organization and evolutionary dynamics. This level of resolution is crucial to assess their potential for horizontal transfer within the hospital setting, where the circulation of highly adaptable clones represents a major epidemiological threat. In addition, characterizing these isolates in such depth provides a unique opportunity to move beyond descriptive surveillance and into translational applications. WGS-based analysis will enable the identification of the most relevant virulence factors associated with urinary tract infections in Honduras, including those that directly contribute to host colonization, immune evasion, or tissue damage. Understanding these genetic traits in a population-specific context may guide the design of novel preventive or therapeutic strategies, either by prioritizing the development of vaccines, antivirulence drugs, or adjuvant therapies that target key determinants of pathogenicity as has been observed in previously published studies focused on other relevant models (Tiwari et al., 2014; Khan et al., 2023; Sohail et al., 2025; Bunduki et al., 2021). Consequently, the genomic dissection of local UPEC populations does not only represent a step toward a more precise understanding of their epidemiology but also opens avenues for the identification of clinically actionable targets that could improve the management of UTIs in vulnerable groups.

## Data Availability

The original contributions presented in the study are included in the article/Supplementary material, further inquiries can be directed to the corresponding author.

## Footnotes

^1^ https://sourceforge.net/projects/gelj/

^2^ https://www.graphpad.com/

^3^ https://jasp-stats.org/download/
