# Supplementary material for: Comprehensive molecular and phenotypic profiling of uropathogenic Escherichia coli in a Honduran healthcare setting: virulence, resistance and phylogeny
Source: Front Microbiol. 2025 Oct 2;16:1656938. doi: 10.3389/fmicb.2025.1656938 (PMC12528171; doi:10.3389/fmicb.2025.1656938)
Supplement: Supplementary file 1 [file Table_1.docx]

Supplementary Material

# Supplementary Figures


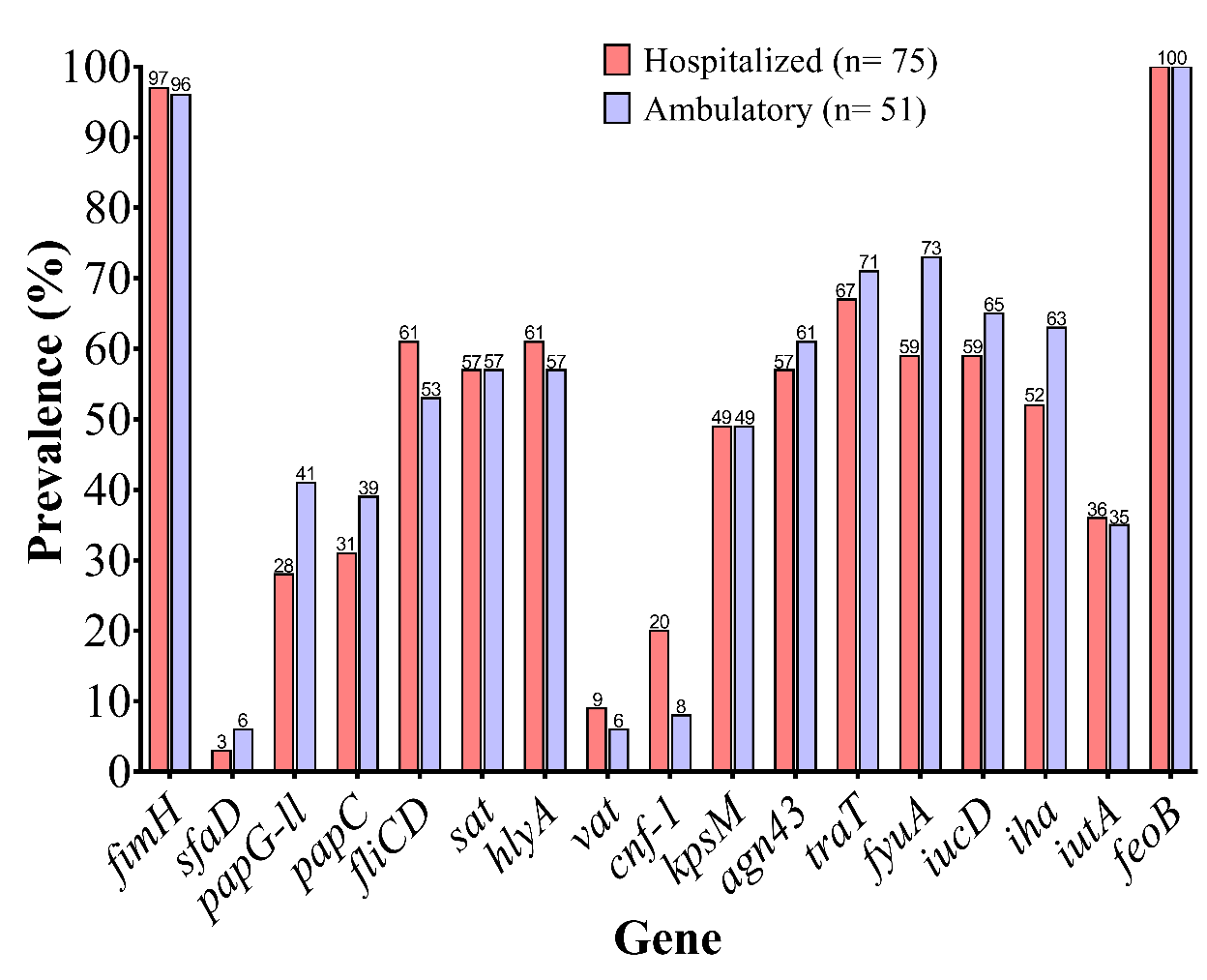


**Supplementary Figure 1.** Prevalence of virulence genes in *Escherichia coli* isolates from hospitalized (n = 75) and ambulatory (n = 51) female patients. The bar chart shows the percentage of isolates carrying each virulence gene. Red bars represent hospitalized patients, and purple bars represent ambulatory patients. *fimH*: fimbrial adhesin of type 1 pilus; *sfaD*/*focC*: S fimbriae minor subunit / F1C fimbriae chaperone; *papG*-II: type P pilus adhesin allele 2; *papC*: type P pilus chaperone; *fliCD*: flagellin subunit / flagellar cap; sat: autotransporter secreted toxin; *hlyA*: α-hemolysin; *vat*: vacuolating autotransporter toxin; *cnf*-1: cytotoxic necrotizing factor 1; *kpsM*: capsular antigen variant; *agn43*: antigen 43; *traT*: serum resistance protein; *fyuA*: yersiniabactin receptor; *iucD*: aerobactin biosynthesis gene; *iha*: bifunctional enterobactin receptor/adhesin protein; *iutA*: ferric aerobactin receptor; *feoB*: ferrous iron transport protein B.


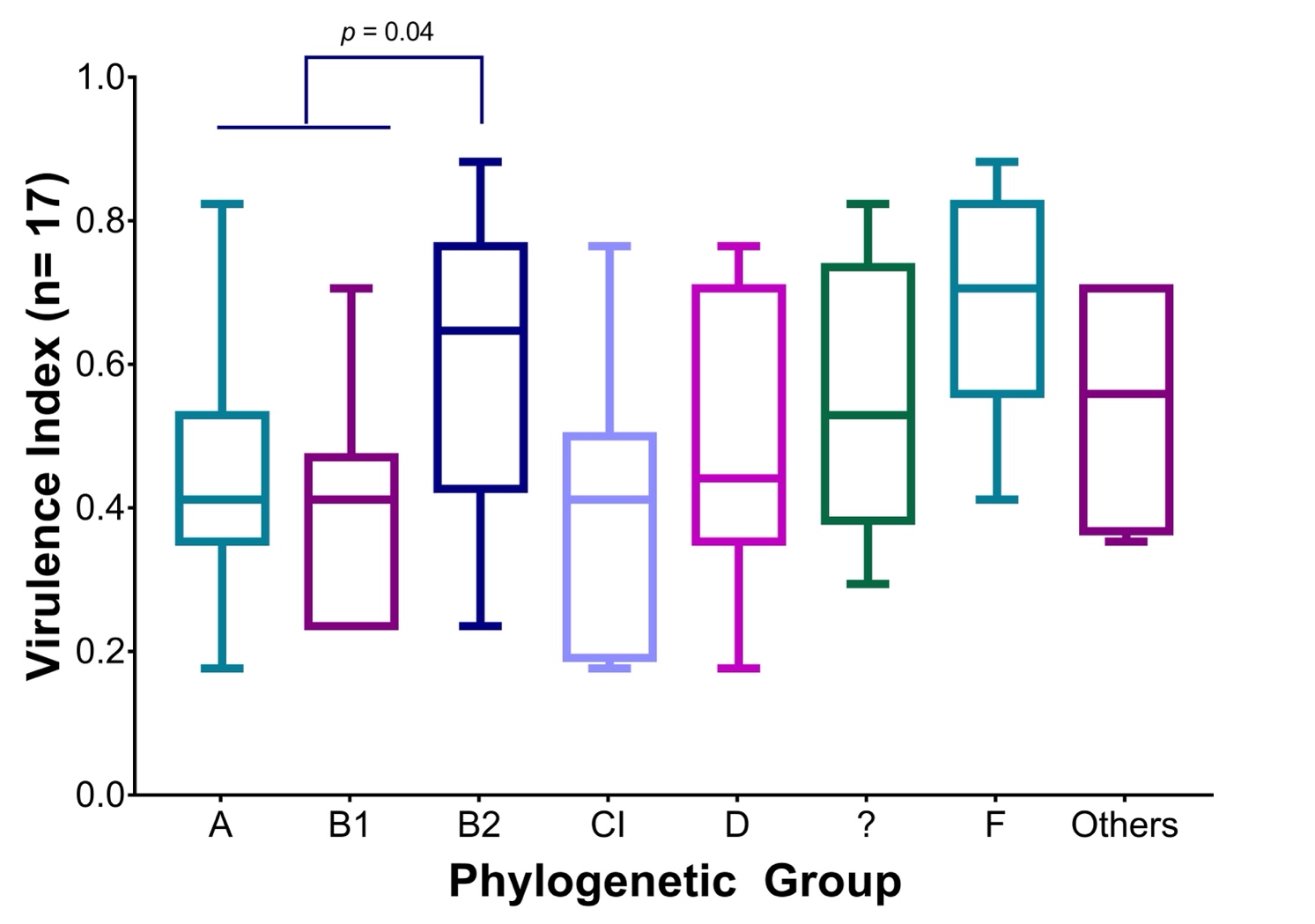


**Supplementary Figure 2.** Distribution of the virulence index in *Escherichia coli* isolates (n = 17) according to their phylogenetic group. The virulence index was calculated as the ratio of detected virulence genes to the total number of genes evaluated. Each box represents the interquartile range (IQR), i.e., the interval between the 25th percentile (Q1) and the 75th percentile (Q3), encompassing the central 50% of the data. The line within the box indicates the median, while the whiskers extend to the minimum and maximum values within 1.5 times the IQR. A statistically significant difference was observed between groups B1 and B2 (*p* = 0.04).


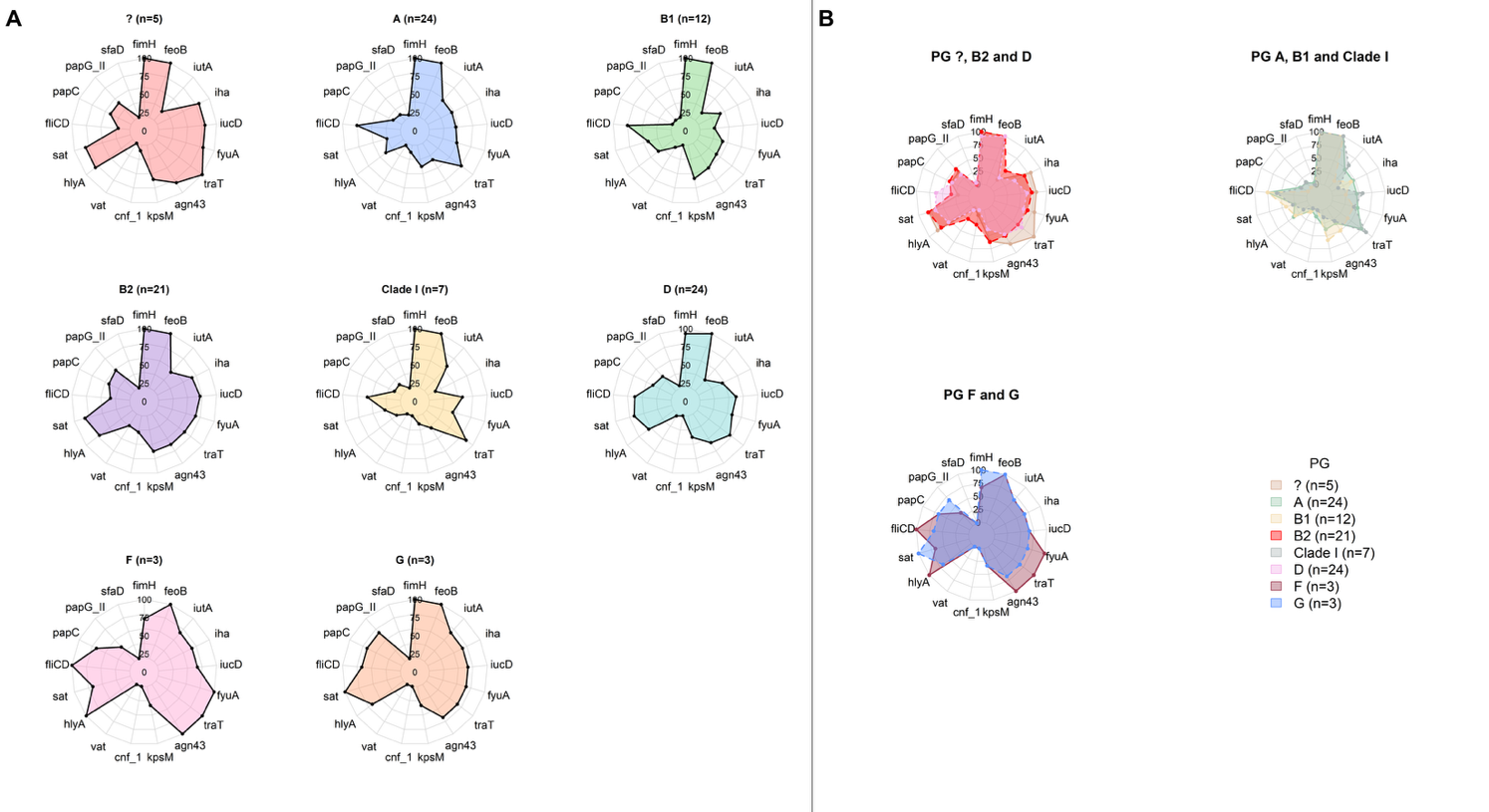


**Supplementary Figure 3.** Virulence and phylogenetic groups in *Escherichia coli* isolates from women patients. **A:** Distribution of virulence genes by phylogenetic group. Each radar chart represents the prevalence of 17 virulence-associated genes within each phylogroup: A, B1, B2, C1, D, F, G, “?”, and Others. **B:** Phylogroups showing similarities in virulence gene distribution patterns. Groups were clustered based on shared virulence profiles.


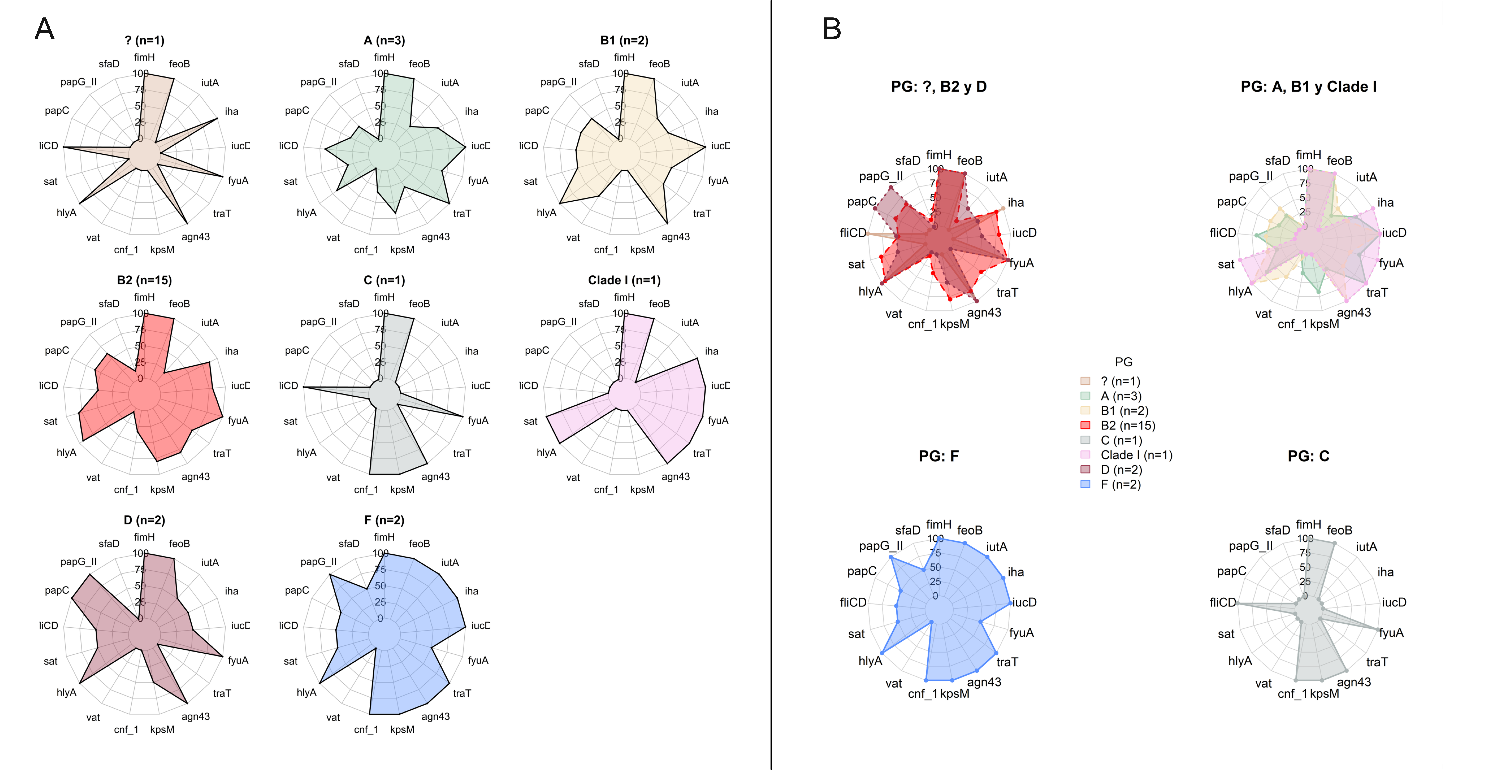


**Supplementary Figure 4.** Virulence and phylogenetic groups in *Escherichia coli* isolates from men. **A:** Distribution of virulence genes by phylogenetic group. Each radar chart represents the prevalence of 17 virulence-associated genes within each phylogroup: A, B1, B2, C, Clade I, D, F, G, and “?”. **B:** Phylogroups grouped according to similarities in virulence gene distribution patterns. Clusters were established based on shared virulence profiles among isolates from men.

**Supplementary Figures 5A–5P**. *Escherichia coli* clinical isolates with the highest similarity, based on clustering analysis of ERIC-PCR banding profiles using the DICE coefficient and UPGMA algorithm with 10% tolerance. The dendrogram (left) reflects genetic relatedness based on these profiles. Columns display the phylogenetic group (PG), detected virulence genes (circle), antibiotic resistance to 18 agents (brown squares), and presence of ESBL and carbapenemase phenotypes (red stars). Panels correspond to the following clinical wards: 5A: Emergency, 5B: Endocrinology, 5C: Gynecology, 5D: Internal Medicine, 5E: Medicine, 5F: External Consultation, 5G: Outpatients, 5H: Pathological Puerperium, 5I: General Medicine, 5J: Pediatrics, 5K: Urology, 5L: Nephrology, 5M: Hematology, 5N: Rheumatology, 5O: Intrahospital Patients, 5P: Outpatients.

**
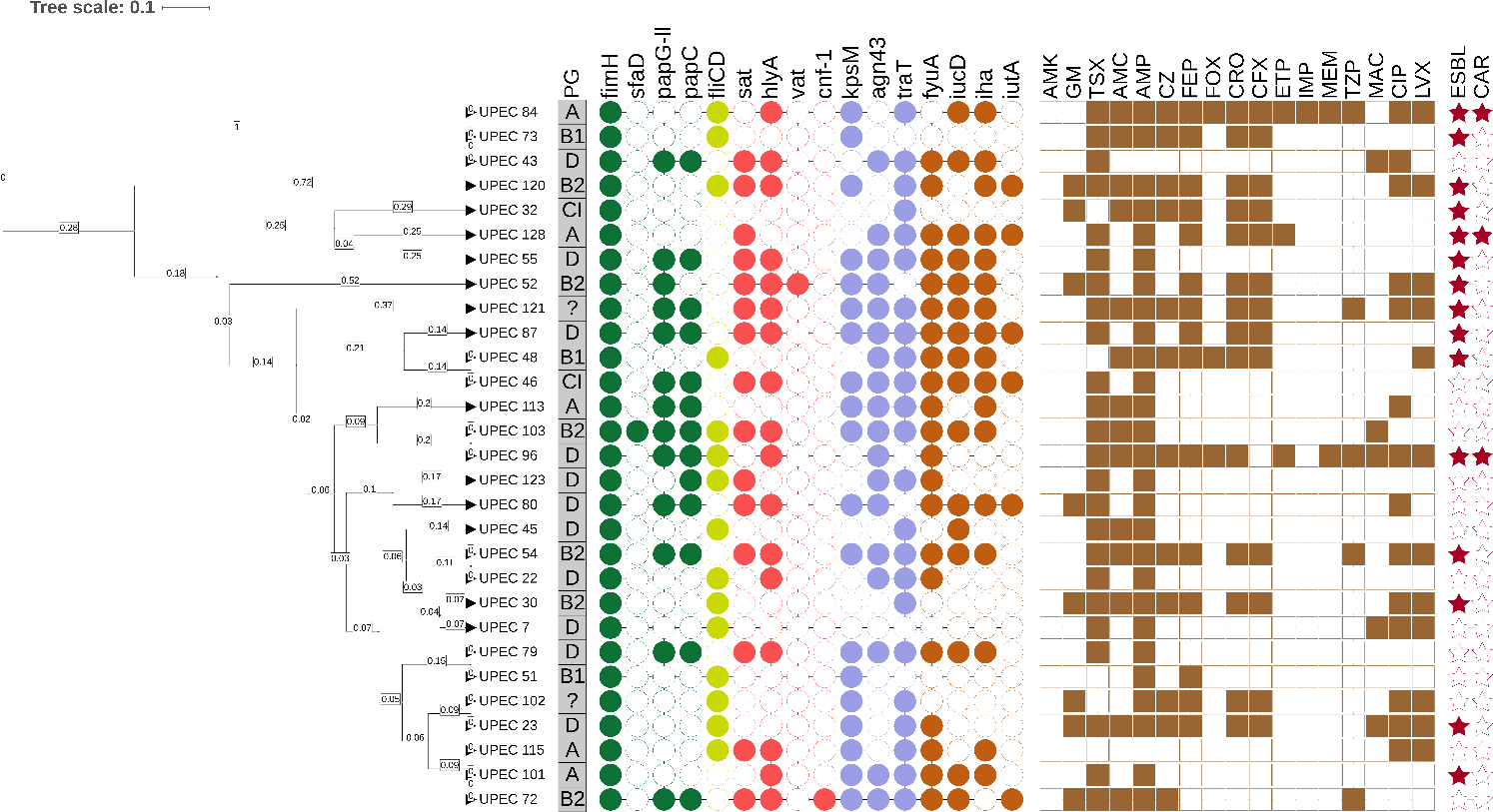
S****upplementary Figure 5A.** *Escherichia coli* clinical isolates from patients in the Emergency ward with the highest similarity, based on clustering analysis, virulence gene profiles, and antibiotic resistance phenotypes.


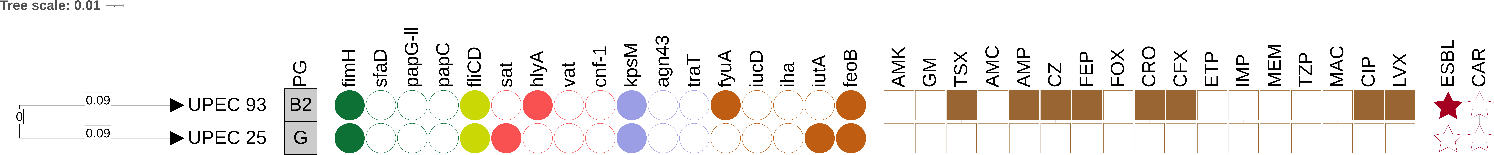


**Supplementary Figure 5B.** *Escherichia coli* clinical isolates from patients in the Endocrinology ward with the highest similarity, based on clustering analysis, virulence gene profiles, and antibiotic resistance phenotypes.


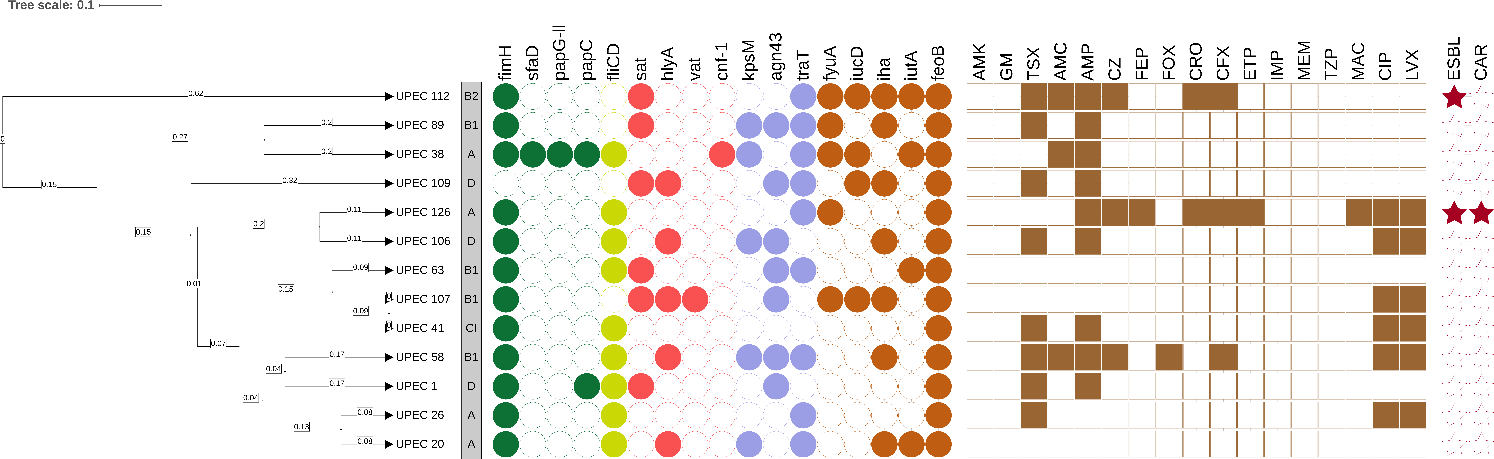


**Supplementary Figure 5C**. *Escherichia coli* clinical isolates from patients in the Gynecology ward with the highest similarity, based on clustering analysis, virulence gene profiles, and antibiotic resistance phenotypes.


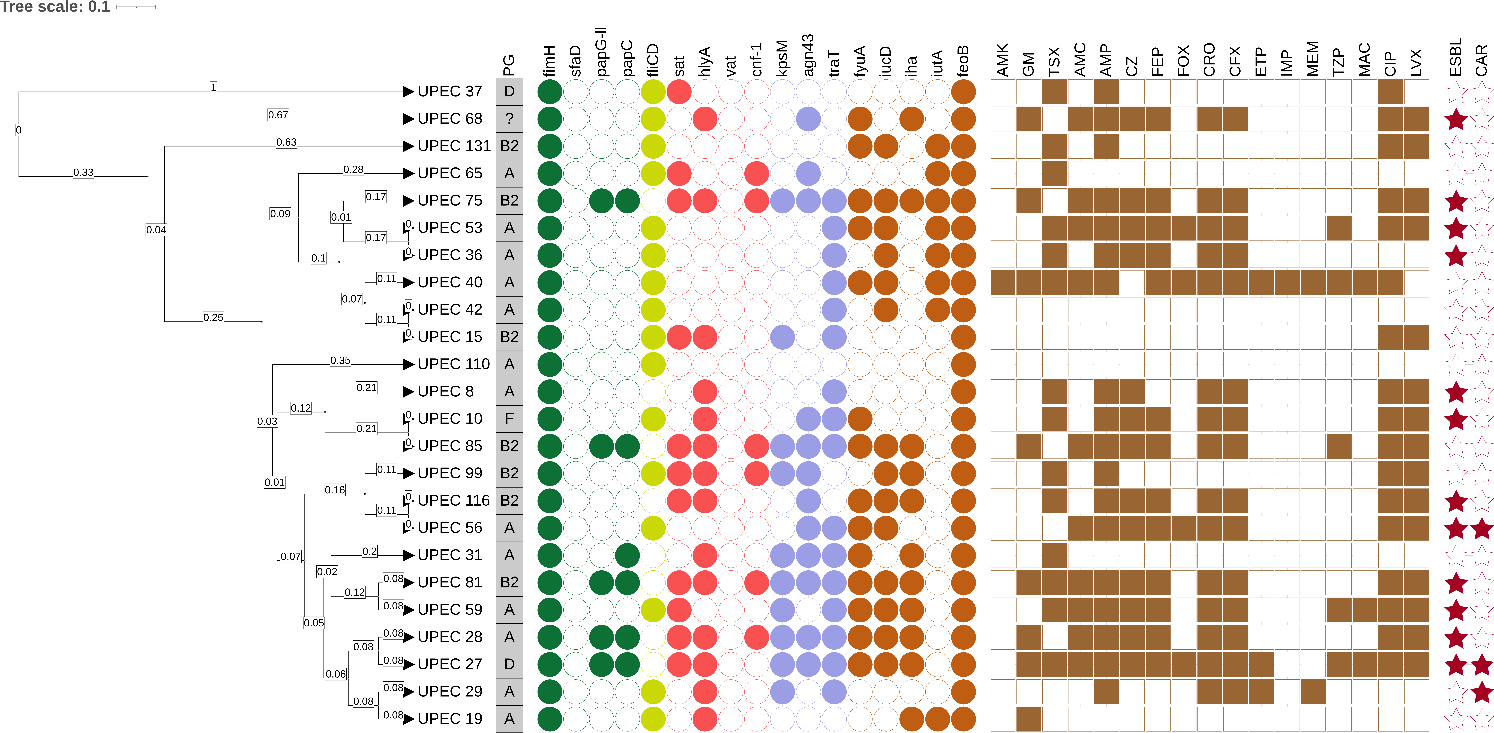
**Supplementary Figure 5D.** *Escherichia coli* clinical isolates from patients in the Internal Medicine ward with the highest similarity, based on clustering analysis, virulence gene profiles, and antibiotic resistance phenotypes.


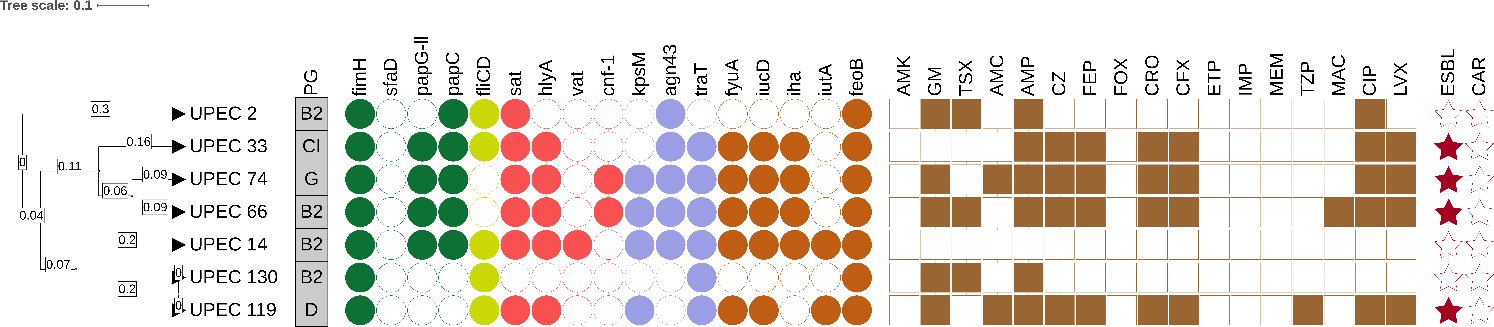


**Supplementary Figure 5E.** *Escherichia coli* clinical isolates from patients in the Medicine ward with the highest similarity, based on clustering analysis, virulence gene profiles, and antibiotic resistance phenotypes.


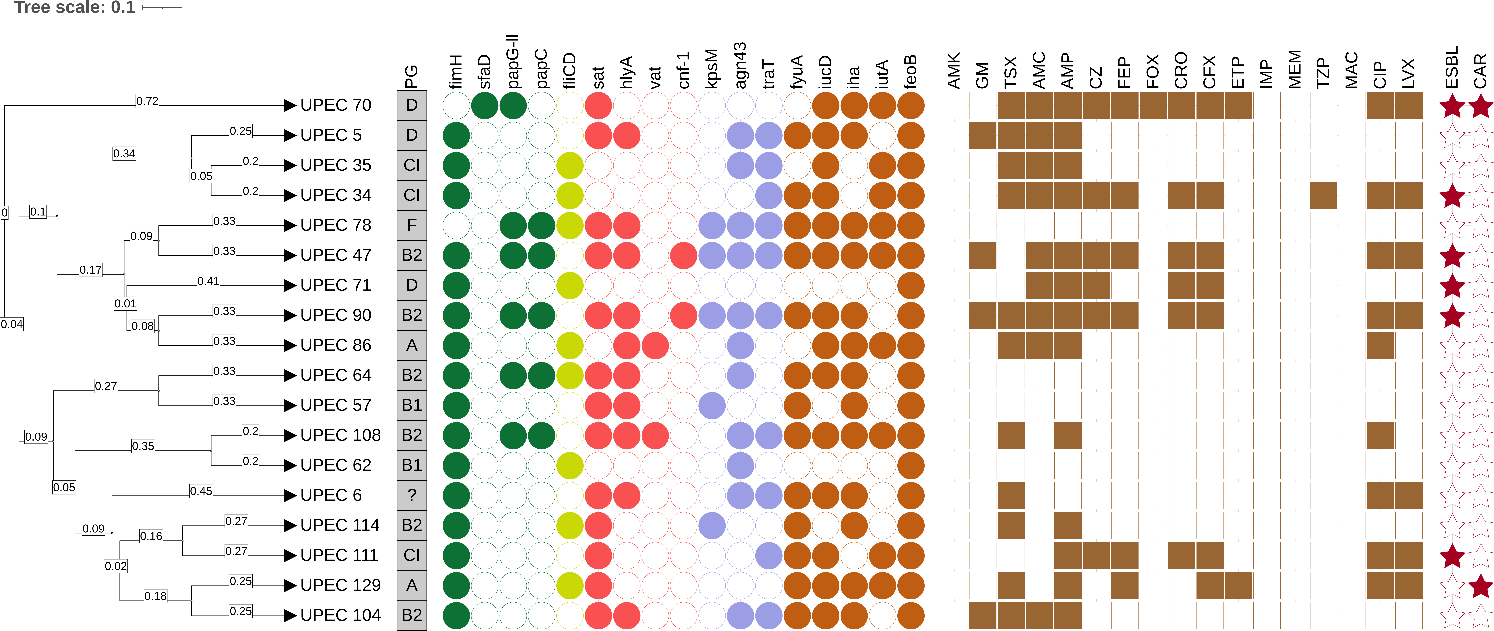


**Supplementary Figure 5F.** *Escherichia coli* clinical isolates from patients in the External Consultation with the highest similarity, based on clustering analysis, virulence gene profiles, and antibiotic resistance phenotypes.


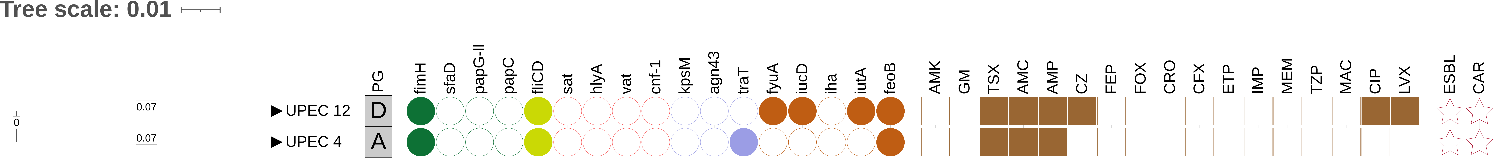


**Supplementary Figure 5G**. *Escherichia coli* clinical isolates from the Outpatients ward with the highest similarity, based on clustering analysis, virulence gene profiles, and antibiotic resistance phenotypes.


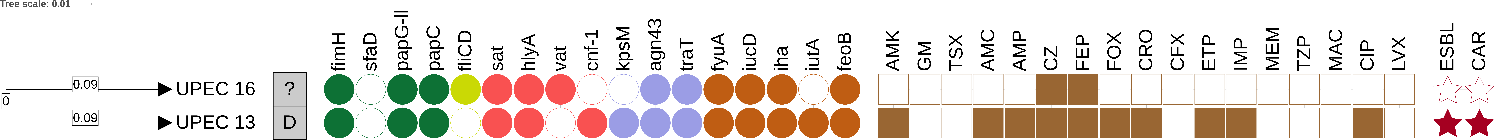


**Supplementary Figure 5H.** *Escherichia coli* clinical isolates from patients in the Pathological Puerperium ward with the highest similarity, based on clustering analysis, virulence gene profiles, and antibiotic resistance phenotypes.


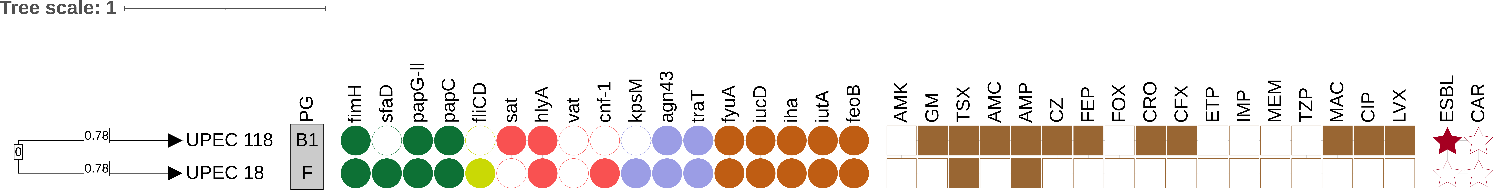


**Supplementary Figure 5I.** *Escherichia coli* clinical isolates from patients in the General Medicine ward with the highest similarity, based on clustering analysis, virulence gene profiles, and antibiotic resistance phenotypes.


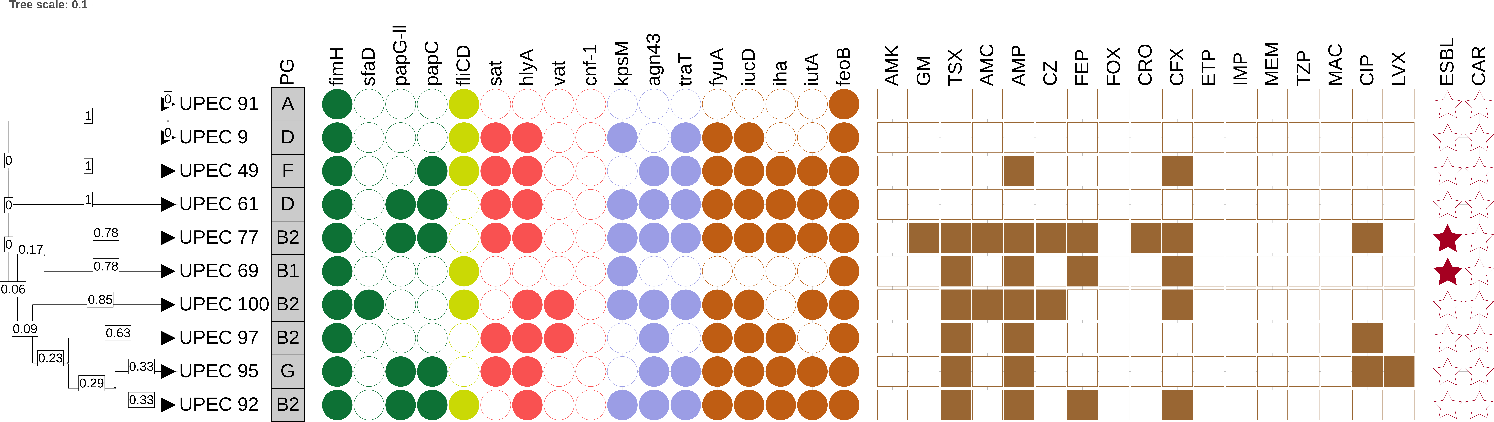


**Supplementary Figure 5J.** Escherichia coli clinical isolates from patients in the Pediatric ward with the highest similarity, based on clustering analysis, virulence gene profiles, and antibiotic resistance phenotypes.


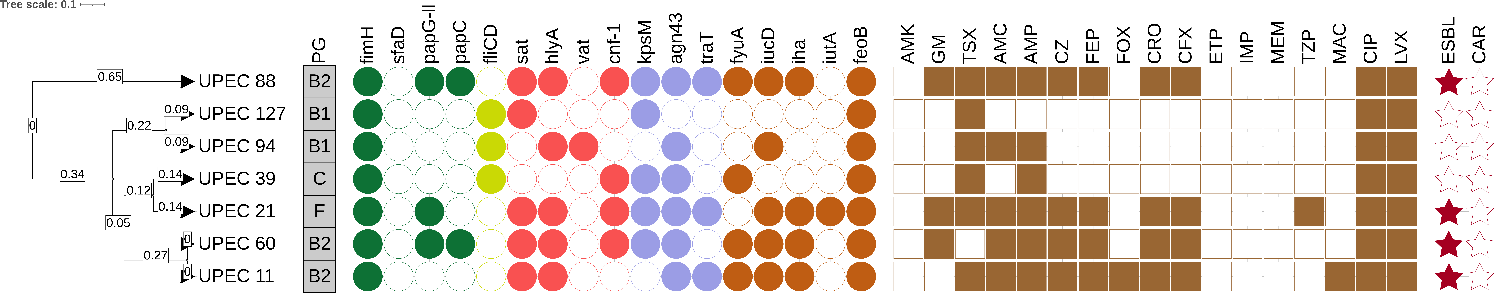


**Supplementary Figure 5K.** *Escherichia coli* clinical isolates from patients in the Urology ward with the highest similarity, based on clustering analysis, virulence gene profiles, and antibiotic resistance phenotypes.


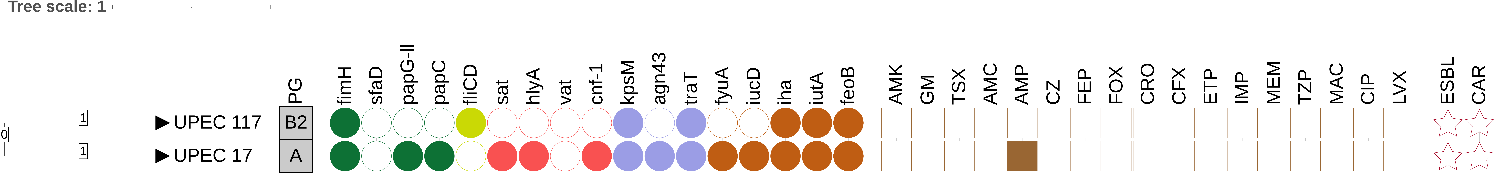


**Supplementary Figure 5L.** *Escherichia coli* clinical isolates from patients in the Nephrology ward with the highest similarity, based on clustering analysis, virulence gene profiles, and antibiotic resistance phenotypes.


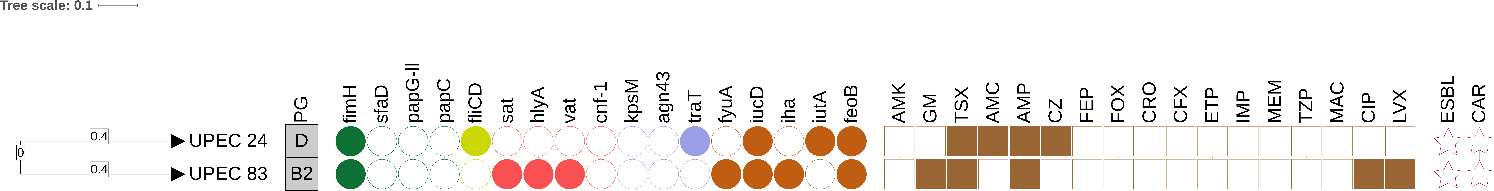


**Supplementary Figure 5M.** *Escherichia coli* clinical isolates from patients in the Hematology ward with the highest similarity, based on clustering analysis, virulence gene profiles, and antibiotic resistance phenotypes.


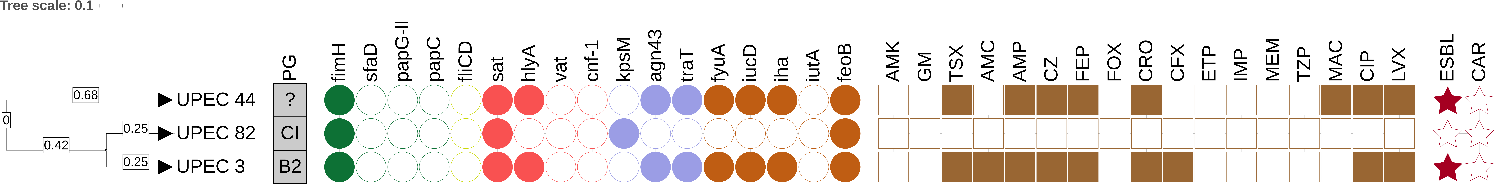


**Supplementary Figure 5N.** *Escherichia coli* clinical isolates from patients in the Rheumatology ward with the highest similarity, based on clustering analysis, virulence gene profiles, and antibiotic resistance phenotypes.


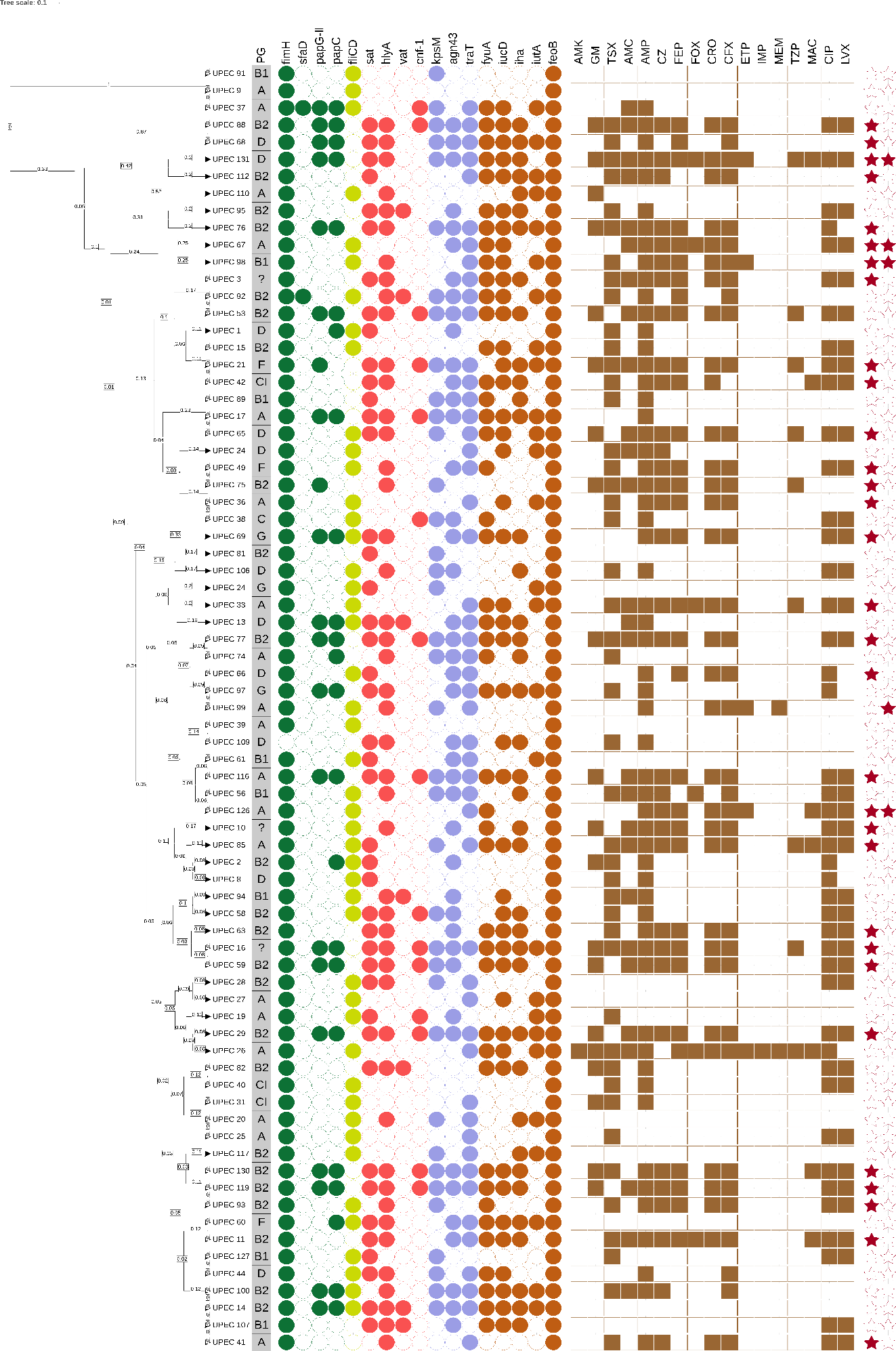


**Supplementary Figure 5O.** *Escherichia coli* clinical isolates from hospitalized patients with the highest similarity, based on clustering analysis, virulence gene profiles, and antibiotic resistance phenotypes.


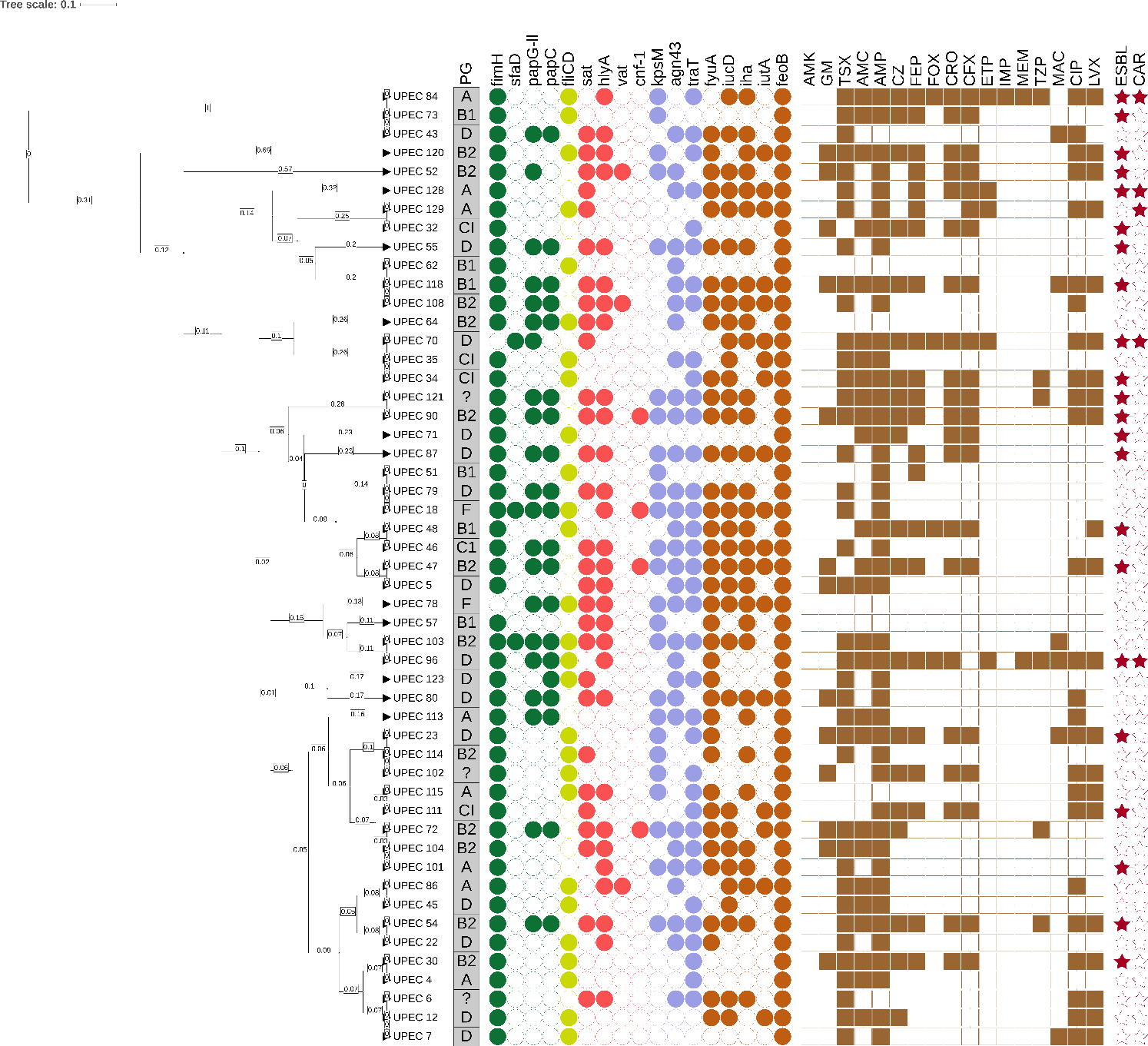


**Supplementary Figure 5P.** *Escherichia coli* clinical isolates from Outpatients with the highest similarity, based on clustering analysis, virulence gene profiles, and antibiotic resistance phenotypes.

**Supplementary Table 1.** List of primers used in this study

**Supplementary Table 1.** Primers used in this study

| **Characteristic** | **Primer Name** | **Product** | **Sequence 5′ to 3′** | **Size product** | **Tm °C** | **Ref** |
| --- | --- | --- | --- | --- | --- | --- |
| Phylogenetic group | AceK.f | *arpA* | AACGCTATTCGCCAGCTTGC | 400 bp | 57 | (1-5) |
|  | ArpA1.r |  | TCTCCCCATACCGTACGCTA |  |  |  |
|  | chuA.1b | *chuaA* | ATGGTACCGGACGAACCAAC | 288 bp |  |  |
|  | chuA.2 |  | TGCCGCCAGTACCAAAGACA |  |  |  |
|  | yjaA.1b | *yjaA* | CAAACGTGAAGTGTCAGGAG | 211 bp |  |  |
|  | yjaA.2b |  | AATGCGTTCCTCAACCTGTG |  |  |  |
|  | TspE4C2.1b | *TspE4C2* | CACTATTCGTAAGGTCATCC | 152 bp |  |  |
|  | TspE4C2.2b |  | AGTTTATCGCTGCGGGTCGC |  |  |  |
|  | ArpAgpE.f | *arpA* | AACGCTATTCGCCAGCTTGC | 301 bp |  |  |
|  | ArpAgpE.r |  | TCTCCCCATACCGTACGCTA |  |  |  |
|  | trpAgpC.1 | *trpA* | AGTTTTATGCCCAGTGCGAG | 219 bp |  |  |
|  | trpAgpC.2 |  | TCTGCGCCGGTCACGCCC |  |  |  |
|  | trpBA.f | *trpA* | CGGCGATAAAGACATCTTCAC | 489 bp |  |  |
|  | trpBA.r |  | GCAACGCGGCCTGGCGGAAG |  |  |  |
|  | ybgD.1 | *ybgD* | TATGCGGCTGATGAAGGATC | 177 bp |  |  |
|  | ybgD.2 |  | GTTGACTAAGCGCAGGTCGA |  |  |  |
|  | cfaB.1 | *cfaB* | CTAACGTTGATGCTGCTCTG | 384 bp |  |  |
|  | cfaB.2 |  | TGCTAACTACGCCACGGTAG |  |  |  |
| Virulence | fimHF | *fimH* | TTATGGCGGCGTGTTATC | 545 bp | 54 | (6)  (6) |
|  | fimHR |  | TCCCTACTGCTCCTAACG |  |  |  |
|  | sfaD/focCF | *sfaD/focC* | AGGCAAATGGACAGGTATGG | 412 bp |  |  |
|  | sfaD/focCR |  | TCACCCAGAACAAACTTTCC |  |  |  |
|  | papG-IIF | *papG-II* | ATTCACCATAGAGGCGACTG | 237 bp |  |  |
|  | papG-IIR |  | ATCATTATGCGGCTCAGAC |  |  |  |
|  | papCF | *papC* | TTCTCTCTCCCTCAATACGG | 925 bp |  |  |
|  | papCR |  | TTATAACCTCAACGGGACGG |  |  |  |
|  | fliCDF | *fliCD* | CCGAATCAGAGTTAGTTCCG | 610 bp | 60 |  |
|  | fliCDR |  | CCCAGCGATGAAATACTTGC |  |  |  |
|  | satF | *sat* | GTTGGCAAACAGGTCAAAC | 809 bp |  |  |
|  | satR |  | CTCGGAGTATTGGCTTCAG |  |  |  |
|  | hlyAF | *hlyA* | GATACGCTGATAGGTGAG | 564 bp | 58 |  |
|  | hlyAR |  | CCAGGTGTGACTCAATAC |  |  |  |
|  | kpsMF | *kpsM* | CCAGAGTAGATATGACCAG | 409 bp |  |  |
|  | kpsMR |  | CTACGAGAAATACGAACAC |  |  |  |
|  | agn43F | *agn43* | CACACAGCCACTAATACC | 488 bp | 58 |  |
|  | agn43R |  | CACCTGAATACCCTTACC |  |  |  |
|  | vatF | *vat* | ATACAGTCTCGTCTCTGG | 670 bp |  |  |
|  | vatR |  | GTGACAGTCCCTTTATCC |  |  |  |
|  | cnf-1F | *cnf-1* | CAGACTCATCTTCACTCG | 551 bp |  |  |
|  | cnf-1R |  | AGACAGAGACCTTACGAC |  |  |  |
|  | traTF | *traT* | TGGTATAGTTCACATCTTCC | 233 bp |  |  |
|  | traTR |  | TAAAGCCTACTACTGGATTC |  |  |  |
|  | fyuAF | *fyuA* | CGCCAGTAAACAATCTTCCC | 937 bp | 60 |  |
|  | fyuAR |  | CCCAAACACCATATCAACGG |  |  |  |
|  | iucDF | *iucD* | CGTGAGACCCAGTTTATTTCC | 334 bp |  |  |
|  | iucDR |  | GGGCTGCTGAAGATATGAATAACC |  |  |  |
|  | iutAF | *iutA* | GTTCACGCTCTTTGTCAGG | 801 bp |  |  |
|  | iutAR |  | GGGCTTAATCTCGGGAAAGG |  |  |  |
|  | ihaF | *iha* | TGTGCTCTGGTTTGATATGG | 594 bp |  |  |
|  | ihaR |  | CATTCTGGGTGCCTTATATCC |  |  |  |
|  | feoBF | *feoB* | GTCTAACCTTGAGCGTAACC | 736 bp |  |  |
|  | feoBR |  | GGCGAGGAAGATAGTCAGC |  |  |  |
| Identification | ybbWF | *ybbW* | TGATTGGCAAAATCTGGCCG | 667 bp |  |  |
|  | ybbWR |  | ATACTGGCAATCAGTACGCC |  |  |  |
| ERIC PCR | ERIC1 | ERIC | AAGTAAGTGACTGGGGTGGGGTGAGCG | Variable | 53 | (7) |
|  | ERIC2 |  | ATGTAAGCTCCTGGGGATTCAC |  |  |  |

**Supplementary material 1A.** Demographic and molecular characterization of clinical *Escherichia coli* isolates associated with urinary tract infections. The dataset includes information on patient gender, age group, and hospital ward of origin. Each isolate was screened for the presence of 17 virulence-associated genes, with results displayed as binary values (1 = gene present, 0 = gene absent). The total number of virulence genes detected per isolate is summarized in the "Genes" column. Distinct virulence profiles are listed in the "Virulence Profile" column, with each unique profile assigned a numerical identifier in the adjacent column. Recurrent profiles are highlighted with the same color to facilitate visual grouping and comparison.

**Supplementary material 1B.** Common virulence profiles in clinical UPEC isolates obtained from female patients. The table displays the virulence gene combinations (column A), the gender of the patient (column B), the codes of clinical isolates sharing each profile (column C), the hospital zones where the samples were collected (column D), and the corresponding age groups of the patients (column E).

**Supplementary material 1C.** Common virulence profiles in clinical UPEC isolates obtained from male patients. The table displays the virulence gene combinations (column A), the gender of the patient (column B), the codes of clinical isolates sharing each profile (column C), the hospital zones where the samples were collected (column D), and the corresponding age groups of the patients (column E).

**Supplementary material 1D.** Pearson’s correlations among all virulence genes evaluated in this study. The table presents Pearson correlation coefficients (r), statistical significance values (p), and 95% confidence intervals (CI) for each gene-gene correlation, calculated from 126 clinical *E. coli* isolates. Significance levels are denoted as follows: **p* < 0.05, ***p* < 0.01, ***p* < 0.001.

**Supplementary material 2A.** Prevalence of antibiotic resistance by gender among clinical UPEC isolates. The table presents the percentage of resistant isolates in male (n = 27) and female (n = 99) patients, along with statistical comparisons using Fisher’s exact test (p-value). For each antibiotic, the odds ratio (OR), relative risk (RR), and their corresponding 95% confidence intervals (CI) are provided. Statistically significant results (p < 0.05) are highlighted in purple. AMK: Amikacin; GM: Gentamicin; TSX: Trimethoprim-Sulfamethoxazole; AMC: Amoxicillin-Clavulanic Acid; AMP: Ampicillin; CZ: Cefazolin; FEP: Cefepime; FOX: Cefoxitin; CRO: Ceftriaxone; CFX: Cefuroxime; ETP: Ertapenem; IMP: Imipenem; MEM: Meropenem; TZP: Piperacillin-Tazobactam; MAC: Nitrofurantoin; CIP: Ciprofloxacin; LVX: Levofloxacin.

**Supplementary material 2B.** Prevalence of antibiotic resistance and ESBL or carbapenemase production by age group and gender among clinical UPEC isolates. The table displays the percentage of resistant isolates in four age groups (<5, 6–12, 13–50, and >50 years old) across the full dataset (n = 126), as well as separately for women (n = 99) and men (n = 27). Percentages are shown for each antibiotic and extended-spectrum β-lactamase (ESBL)- and carbapenemase (CAR)-producing isolates. AMK: Amikacin; GM: Gentamicin; TSX: Trimethoprim-Sulfamethoxazole; AMC: Amoxicillin-Clavulanic Acid; AMP: Ampicillin; CZ: Cefazolin; FEP: Cefepime; FOX: Cefoxitin; CRO: Ceftriaxone; CFX: Cefuroxime; ETP: Ertapenem; IMP: Imipenem; MEM: Meropenem; TZP: Piperacillin-Tazobactam; MAC: Nitrofurantoin; CIP: Ciprofloxacin; LVX: Levofloxacin.

**Supplementary material 2C.** Pearson’s correlations between individual antibiotic resistance, ESBL, and carbapenemase production among clinical UPEC isolates (n = 126). The table presents Pearson correlation coefficients (r), p-values, and 95% confidence intervals (CI) for pairwise comparisons between resistance to specific antibiotics and the presence of ESBL or carbapenemase production. Only positive correlations were tested (one-tailed). Statistically significant correlations are indicated as follows: **p* < 0.05, ***p* < 0.01, ***p* < 0.001.

**Supplementary material 3.** Prevalence of UPEC virulence genes by phylogenetic group. The table shows the percentage of positive isolates, p-values (Fisher’s exact test), odds ratios (OR), relative risk (RR), and 95% confidence intervals (CI) for the presence of 17 virulence genes across different Escherichia coli phylogenetic groups. Comparisons were made between each phylogroup and the rest of the dataset. Statistically significant results showing lower gene prevalence in a specific phylogenetic group are highlighted in green, while those showing higher prevalence are highlighted in purple. *fimH*: Fimbrial adhesin of type 1 pilus*; sfaD*: S fimbriae minor subunit / *F1C* fimbriae chaperone; *papC*: Type P pilus chaperone; *papG-II*: Type P pilus adhesin allele 2; *fliCD*: Flagellin subunit / flagellar cap; *hlyA*: α-hemolysin; sat: Autotransporter secreted toxin; *vat*: Vacuolating autotransporter toxin; *cnf-1*: Cytotoxic necrotizing factor 1; *kpsM*: Capsular antigen variant; *traT*: Serum resistance protein; *agn43*: Antigen 43; *iucD*: Aerobactin biosynthesis gene; *fyuA*: Yersiniabactin receptor; *iha*: Bifunctional enterobactin receptor/adhesin protein; *iutA*: Ferric aerobactin receptor; *feoB*: Ferrous iron transport protein B. CI: Confidence Interval; NA: Not available.

**References for the primers used in this study**

1. Clermont O, Christenson JK, Denamur E, Gordon DM. The C lermont Escherichia coli phylo‐typing method revisited: improvement of specificity and detection of new phylo‐groups. Environmental microbiology reports. 2013;5(1):58-65.

2. Clermont O, Bonacorsi S, Bingen E. Rapid and Simple Determination of the Escherichia coli Phylogenetic Group. Applied and Environmental Microbiology. 2000;66(10):4555-8.

3. Clermont O, Bonacorsi S, Bingen E. Characterization of an anonymous molecular marker strongly linked to Escherichia coli strains causing neonatal meningitis. Journal of clinical microbiology. 2004;42(4):1770-2.

4. Clermont O, Dixit OV, Vangchhia B, Condamine B, Dion S, Bridier‐Nahmias A, et al. Characterization and rapid identification of phylogroup G in Escherichia coli, a lineage with high virulence and antibiotic resistance potential. Environmental microbiology. 2019;21(8):3107-17.

5. Clermont O, Lescat M, O'Brien CL, Gordon DM, Tenaillon O, Denamur E. Evidence for a human‐specific Escherichia coli clone. Environmental Microbiology. 2008;10(4):1000-6.

6. Ballesteros-Monrreal MG, Arenas-Hernández MM, Barrios-Villa E, Juarez J, Álvarez-Ainza ML, Taboada P, et al. Bacterial morphotypes as important trait for uropathogenic E. coli diagnostic; a virulence-phenotype-phylogeny study. Microorganisms. 2021;9(11):2381.

7. Movahedi M, Zarei O, Hazhirkamal M, Karami P, Shokoohizadeh L, Taheri M. Molecular typing of Escherichia coli strains isolated from urinary tract infection by ERIC-PCR. Gene Reports. 2021;23:101058.
